# Cumulative dose responses for adapting biological systems

**DOI:** 10.1098/rsif.2024.0877

**Published:** 2025-08-13

**Authors:** Ankit Gupta, Eduardo Sontag

**Affiliations:** ^1^ Department of Biosystems Science and Engineering, ETH Zürich, Basel, Switzerland; ^2^ Departments of Electrical and Computer Engineering and Bioengineering, Northeastern University—Boston Campus, Boston, MA, USA; ^3^ Laboratory for Systems Pharmacology, Harvard Medical School, Boston, MA, USA; ^4^ LIDS, MIT, Cambridge, MA, USA

**Keywords:** dose response, perfect adaptation, systems biology, incoherent feedforward loops, integral feedback, immunology, area under the curve

## Abstract

Physiological adaptation is a fundamental property of biological systems across all levels of organization, ensuring survival and proper function. Adaptation is typically formulated as an asymptotic property of the *dose response (DR*), defined as the level of a response variable with respect to an input parameter. In pharmacology, the input could be a drug concentration; in immunology, it might correspond to an antigen level. In contrast to the DR, this paper develops the concept of a transient, finite-time, cumulative dose response (cDR)*,* which is obtained by integrating the response variable over a fixed time interval and viewing that integral—area under the curve—as a function of the input parameter. This study is motivated by experimental observations of cytokine accumulation under T-cell stimulation, which exhibit a non-monotonic cDR. It is known from the systems biology literature that only two types of network motifs, incoherent feedforward loops and negative integral feedback (IFB) mechanisms, can generate adaptation. Three paradigmatic such motifs—two types of incoherent loops and one integral feedback—have been the focus of much study. Surprisingly, it is shown here that these two incoherent feedforward loop motifs—despite their capacity for non-monotonic DR—always yield a monotonic cDR, and are therefore inconsistent with these experimental data. On the other hand, this work reveals that the IFB motif is indeed capable of producing a non-monotonic cDR, and is thus consistent with these data.

## 1. Introduction

The capability to adapt and to formulate appropriate responses to environmental cues is a key factor for the survival of life, at every level from individual cells, to organisms, to societies [1–4]. A delicate balance is needed in this process: organisms maintain tightly regulated levels of vital quantities, even in the face of variations to be counteracted upon, a property sometimes called homeostasis or adaptation, all the while being able to detect and react to changes in the environment. Underlying adaptation at the cellular level are dynamical signal transduction and gene regulatory networks that measure and process external and internal chemical, mechanical and physical conditions (ligand, nutrient, oxygen concentrations, pressure, light, and temperature) eventually leading to changes in metabolism, gene expression, cell division, motility, and other characteristics. These mechanisms enable organisms to display transient responses that gradually return to a baseline activity level when presented with relatively constant input stimulation, a phenomenon usually called ‘perfect’ or ‘exact’ adaptation [5].

### 1.1. Dose response and cumulative dose response

**Figure 1.**
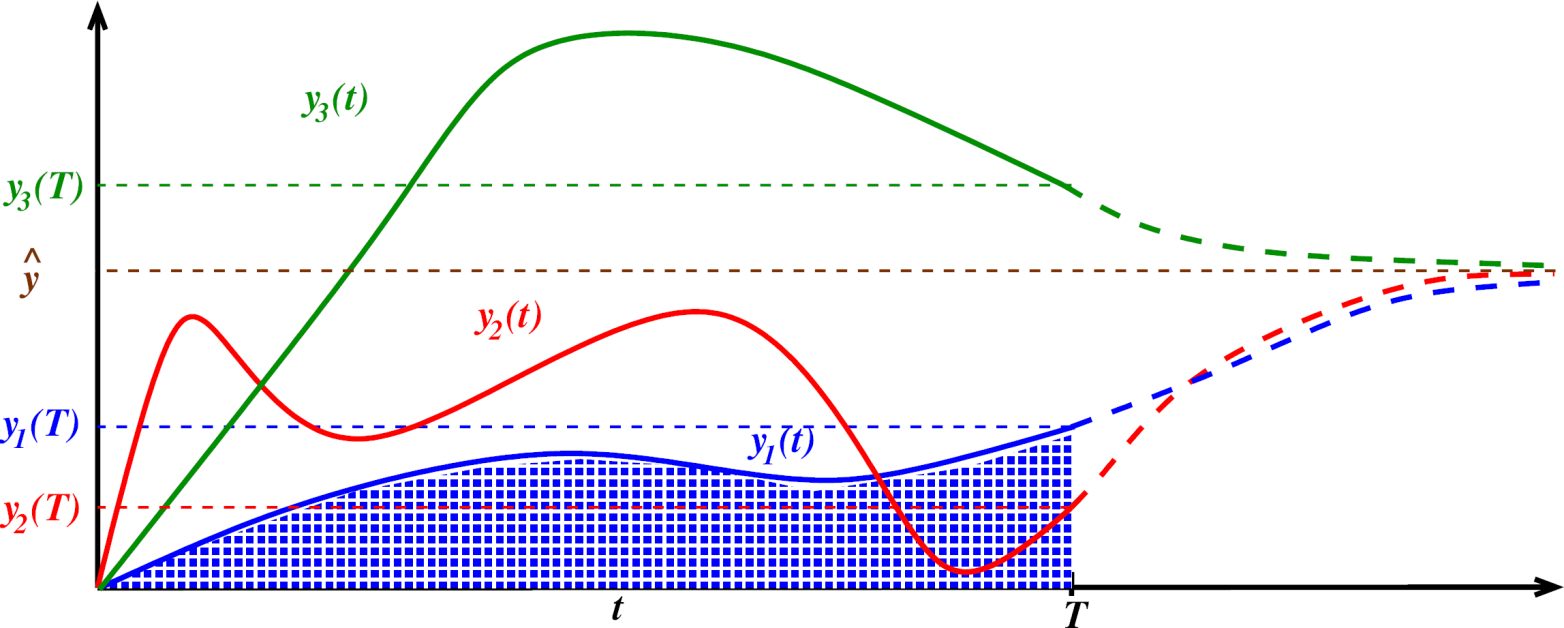
Response functions y(t) plotted against time. As an illustration, three outputs $y_{1}(t)$, $y_{2}(t)$ and $y_{3}(t)$ are shown, corresponding to three inputs $u_{1}$, $u_{2}$ and $u_{3}$, respectively, and their values at a specified time T are shown on the vertical axis. In an adaptive system, the values of all the $y_{i}(t)$'s converge to the same value $\hat{y}$ when t→∞. For each $y_{i}(t)$, solid curves are used for behaviours until the specified time t=T, and dashed lines for the continuation to t=∞. The area of the shaded region represents the integral $\int_{0}^{T}y_{1}(t)\;dt$.

In this article, we continue the study of adaptation mechanisms, with an emphasis on monotonicity properties of an output or reporter variable as a function of an input. We are concerned with ordinary differential equation (ODE) systems that model the interactions of several species, and where there is an ‘input’ (which might represent the dose of a drug or of a genetic inducer), whose level is quantified by the variable u, and an ‘output’ that is time dependent, and whose magnitude at time t is represented by y(t). The input u will be assumed to be constant, and we write $y_{u}(t)$ to highlight the dependence of the output on both the input and time. Figure 1 shows three typical responses that one might observe experimentally (in the figure, we write $y_{i}(t)$ instead of $y_{u_{i}}(t)$, in order to simplify notations).

The dependence on the initial state will not be indicated explicitly; the initial values of all the species will be fixed at values to be discussed.

One defines (‘perfect’) adaptation to constant inputs as the property that, no matter what is the actual value of the input, the numerical value of $y_{u}(t)$ for large t is the same, that is, $\lim_{t\to \infty }\;y_{u}(t)=\hat{y}$ for some fixed value $\hat{y}$ which does not depend on the particular input u (see an illustration in figure 1). This value represents a habituated or no-response state, as one achieves when presented with a constant background noise or level of light. In engineering terms, an adapting system is a ‘high pass filter’ that essentially acts on a derivative of the input. Adaptation, by definition, is an *asymptotic* property, since it ignores finite-time behaviour. On the other hand, *transient* behaviours, particularly how $y_{u}(T)$ varies with u at a fixed time T, are often of interest (What is bethe concentration of a drug in a tumour microenvironment, after 1 h, as a function of the drug dose? What is the size of a tumour after 60 days of the start of therapy, as function of the drug dose? What is the size of the pool of infected individuals in an epidemic population model, as a function of transmission parameters?).

**Figure 2.**
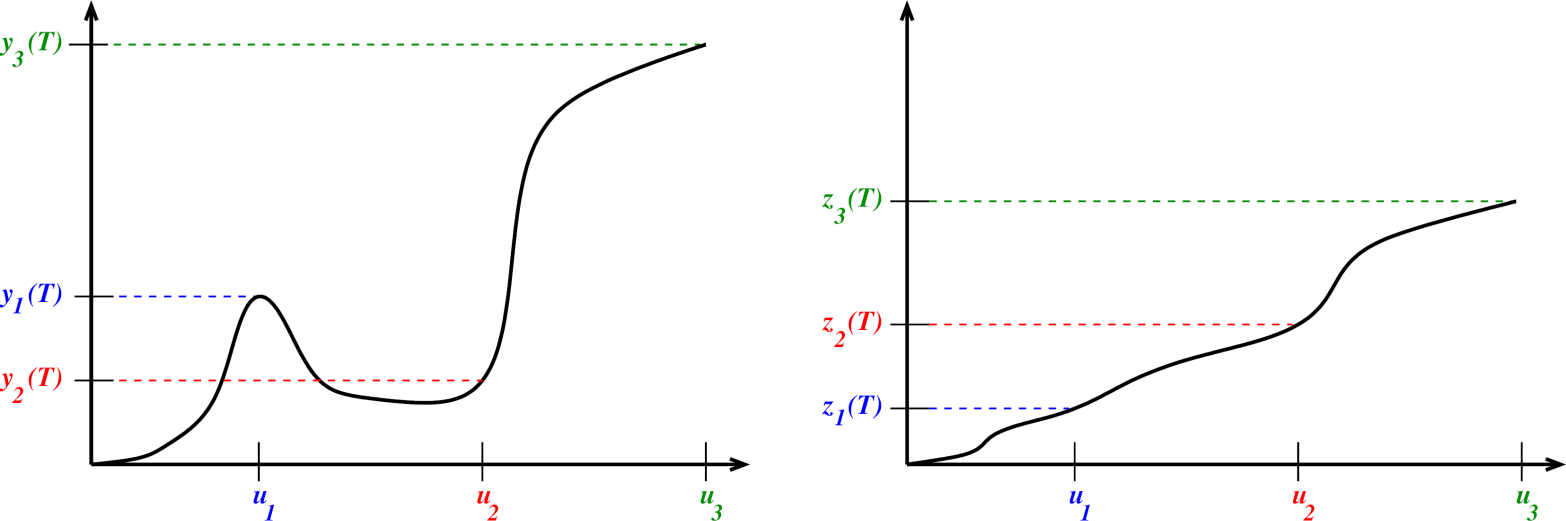
Left: Dose response (DR) at time T, obtained from time-resolved data as in figure 1. Again, the vertical axis represents the evaluation of responses y(t) at a specific time t=T, but now these values are plotted against the respective input u, instead of against time. In this instance, DR is not a monotonic function; for example, $u_{1}<u_{2}$ but $y_{1}(T)>y_{2}(T)$. Right: Cumulative dose response (cDR) at time T; now the vertical axis shows the integral (area under the curve) $z(t)=\int_{0}^{T}y(t)\;dt$ of the response, again plotted against the input u. For example, $z_{1}(t)$ is the area shaded in blue in figure 1. This particular cDR is monotonic. For example, $u_{1}<u_{2}<u_{3}$ and $z_{1}(T)<z_{2}(T)<z_{3}(t)$, because in figure 1 the area under the red curve $y_{2}(t)$ is larger than the area under the blue curve $y_{1}(t)$, even though the final value $y_{2}(T)$ is smaller than $y_{1}(T)$.

We will call $y_{u}(T)$, viewed as a function of u the *dose response* (*DR*) of the given system, and denote it as DR(u,T). One may perform experiments, exciting a system with different input values u and measure $y_{u}(T)$ as the final output value, thus obtaining a plot of y(T) versus u. The left panel of figure 2 shows several DRs obtained from time-resolved data such as presented in figure 1.

If the turnover of y(t) is slow, the molecules or other objects represented by $y_{u}(t)$ may accumulate, for instance, in a particular tissue or the bloodstream. It is often the case that one can only measure experimentally, and that a phenotypical response only depends on, the accumulated value ‘or integral under the curve’ (AUC) $\int_{0}^{T}y_{u}(t)\text{d}t$, which we call the cumulative dose response and denote by cDR(u,T). The right panel of figure 2 shows several cDRs obtained from simulation data as in figure 1. Section 1.9 briefly reviews several areas of biology where cDR’s appear naturally. Specifically, however, this article was motivated by our previous research in immunology that measured cDR’s [6]. We discuss that motivation next.

### 1.2. Motivation for this work: T-cell recognition

Adaptation is central to immunity. In particular, T cells must react to stimulation by pathogens and cancers, yet limit their response to maintain self-tolerance and avoid autoimmune reactions. T-cell activation is triggered by the binding to T-cell receptors (TCRs) to peptide major-histocompatibility complex (pMHC) antigens. Activation results in the production of signalling molecules (cytokines), which in turn may recruit other immune components.

**Figure 3.**
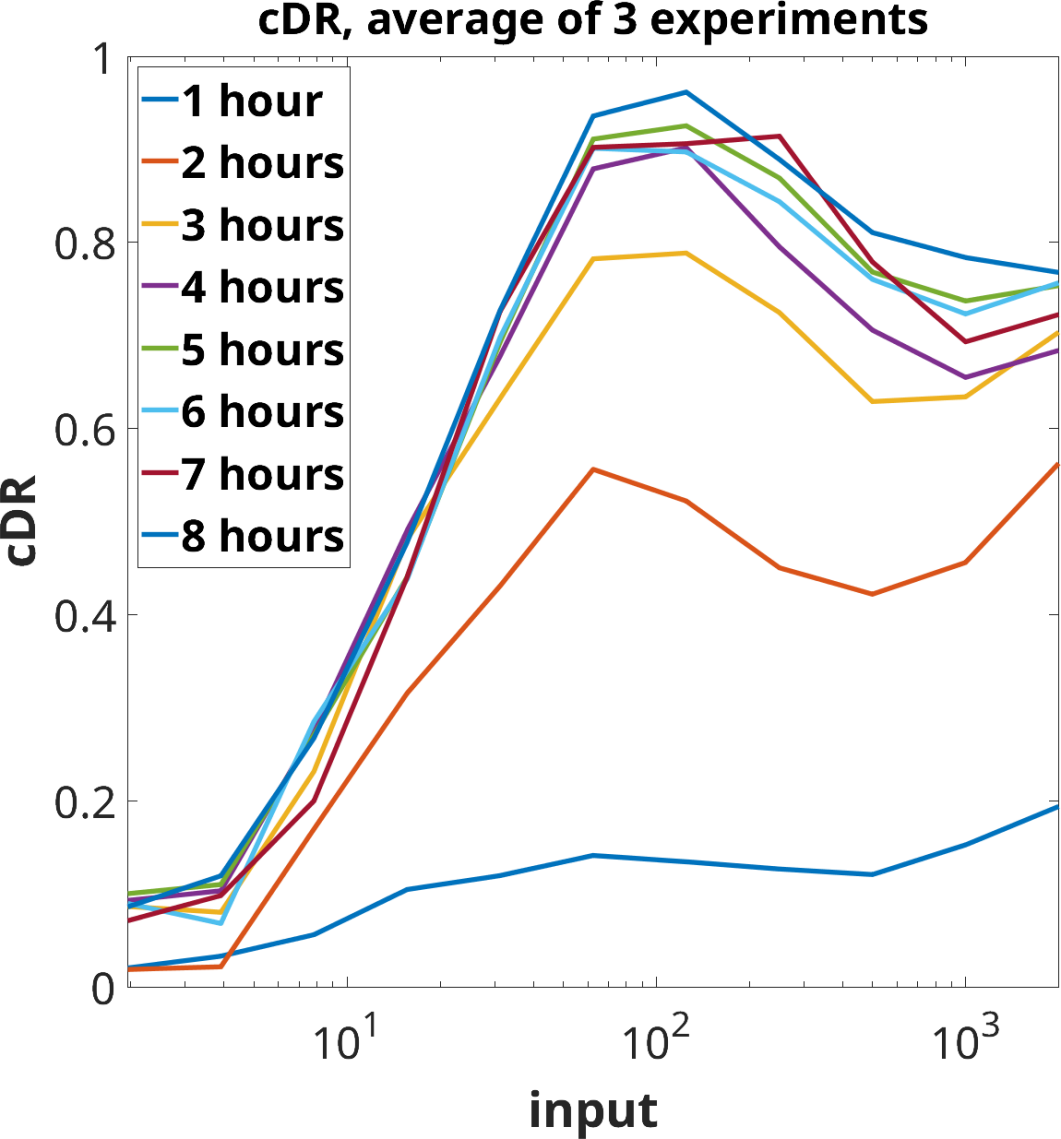
Cumulative dose response based on average of three experiments. Plot uses experimental data from [6] (see also fig. 2B in that paper). Horizontal axis denotes concentrations of the input (in units of ligand in ng/well).

The study in [6] examined the response of immune CD8^+^ T cells to external antigen inputs, demonstrating perfect adaptation across a wide range of antigen affinities. Specifically, the experiments in [6] involved stimulating primary human CD8^+^ T cells (with the c58c61 TCR) with recombinant pMHC antigen 9V immobilized on plates, which served as the input u at various constant concentrations. This antigen is a cancer peptide routinely used in studies of T-cell binding and antigen discrimination. The cumulative amount of cytokine TNF-α (the output y(t)) secreted into the culture medium was measured. Figure 3 shows the cDR when one averages the results of three biological replicates. It shows the average cumulative TNF-α abundance ($z(t)=\int_{0}^{t}y(s)\;\text{d}s$) plotted against several constant concentrations u, measured at various times (t=1 to 8 h). Note the non-monotonic, and even somewhat oscillatory, behaviour.

The work in [6] thus raises the question of what network motifs are capable, at least for suitable parameters, to exhibit perfect adaptation as well as non-monotonic cDR as seen in these experiments. It was speculated, on the basis of numerical exploration, that incoherent feedforward loops (IFFLs) cannot result in non-monotonic cDR and thus cannot explain T-cell adaptation as measured by accumulated cytokines, unless a thresholding mechanism is imposed.

Our main results in this article confirm in a mathematically rigorous way that, indeed, the main two common types feedforward loops (called ‘IFFL1’ and ‘IFFL2’ below) can *never* exhibit such behaviours, because their cDR’s are *always monotonic*. This is especially surprising for one of them (‘IFFL1’) because for such systems the DR itself can be non-monotonic, yet the cDR is monotonic, as in the cartoon illustrations in figure 2. To see this with an example, consider the following system of two differential equations, which is a particular example of the general equations (1.5) and (1.6) discussed later,


(1.1)x˙=−x+u(1.2)y˙=−10xy+u.

**Figure 4.**
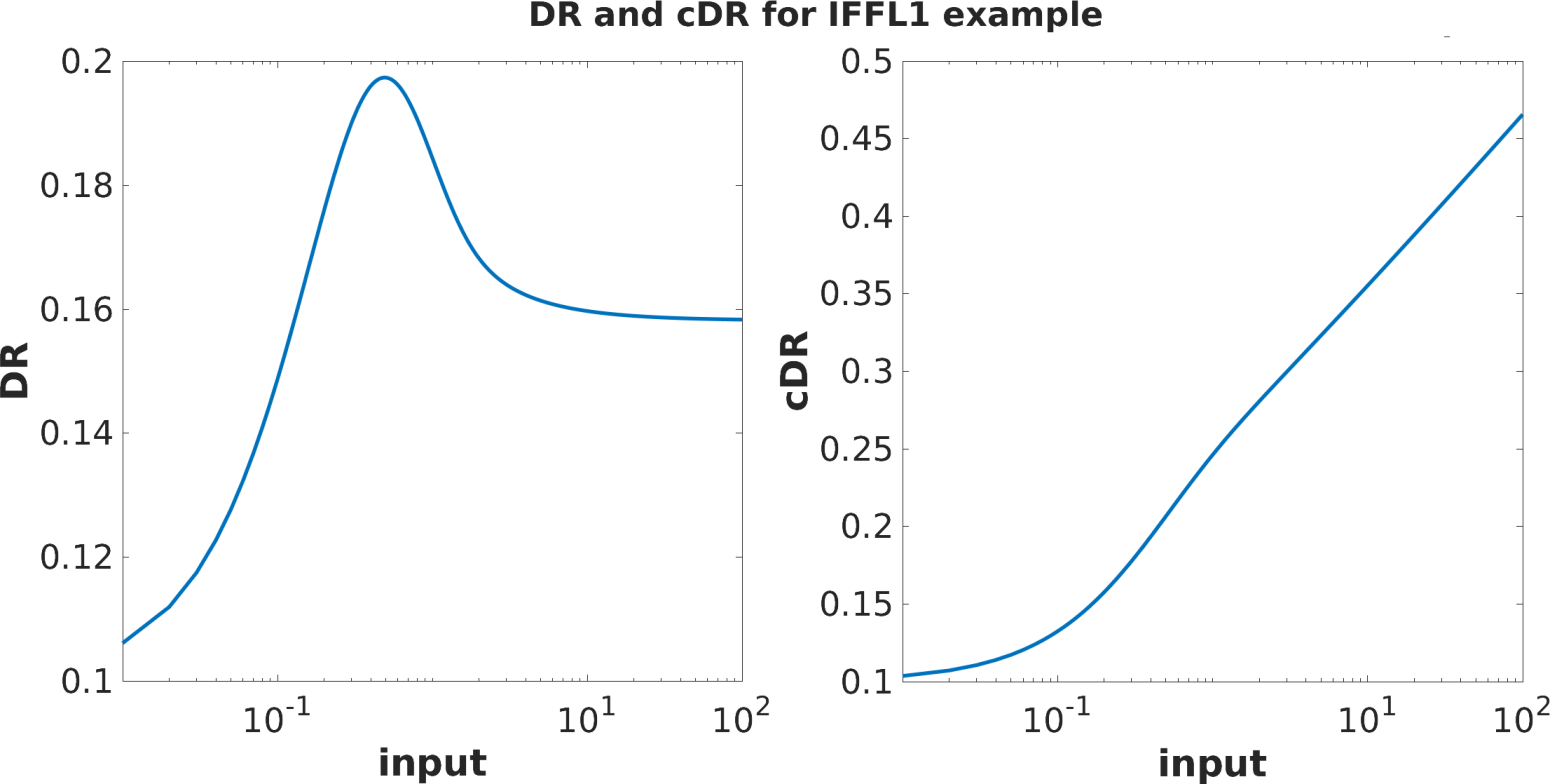
Plots of DR (y(t)) and cDR ($\int_{0}^{T}y(t)dt$) for the example in equations (1.1) and (1.2) . The initial conditions are x(0)=0, y(0)=1/10, and the time horizon is T=1.5. Using logarithmic scale on inputs, for comparison with experimental plots. Observe that the DR is non-monotonic, yet, surprisingly, the cDR is monotonic. Our main theorem proves monotonicity of the cDR in general, for all IFFL1 and IFFL2 systems.

This is an adapting system: for any given constant input u>0, the steady states are $\bar{x}=u$ and $\bar{y}=1/10$, which is independent of u, so that the steady-state output $\hat{y}=1/10$ is independent of u. Figure 4 shows plots of the DR (non-monotonic) and the cDR (monotonic).

We complement the result for feedforward loops with the new finding that, on the other hand, the standard *nonlinear integral feedback* for adaptation (‘IFB’ below) is indeed capable of showing non-monotonic cDR, and thus is potentially a mechanism that is consistent with the experimentally observed non-monotonic cDR. To see this with an example, consider the following system of two differential equations, which is a particular example of the general equations (1.9) and (1.10) discussed later,


(1.3)x˙=x(y−6),(1.4)y˙=ux−y.

**Figure 5.**
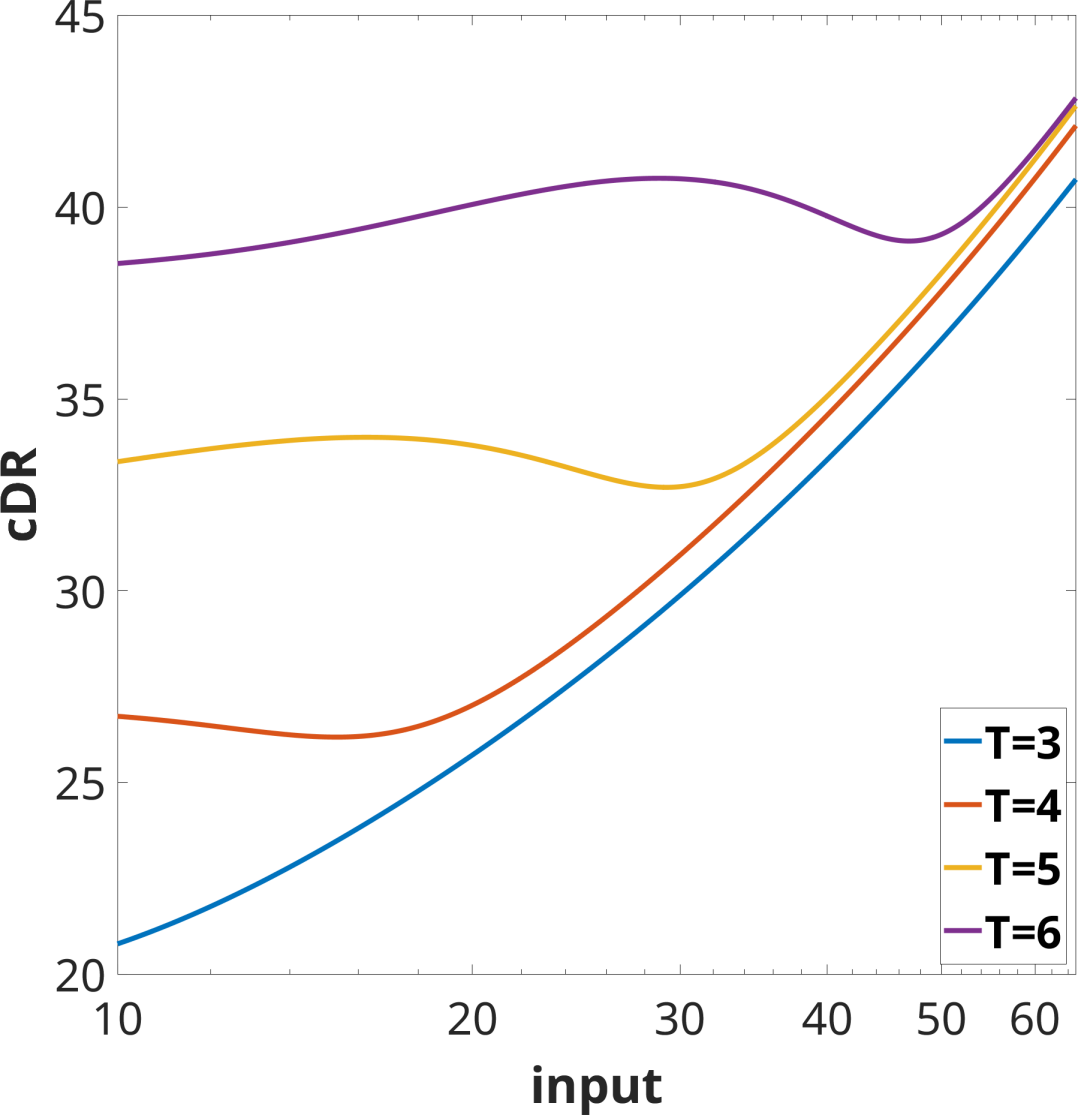
Plot of cDR ($\int_{0}^{T}y(t)dt$) for the IFB example in equations (1.3) and (1.4). The initial conditions are x(0)=0.1, y(0)=6, and time horizons shown are T=3,4,5,6. Observe that, just as with the experimental data plotted in figure 3, the cDR is more monotonic (on the shown ranges, at least) for smaller time horizons T. Using logarithmic scale on inputs, for comparison with experimental plots.

This is also an adapting system: for any given constant input u>0, the steady states are $\bar{x}=u/6$ and $\bar{y}=6$, so that the steady-state output $\hat{y}=6$ is independent of u. Figure 5 shows plots of the (non-monotonic) cDR.

**Figure 6.**
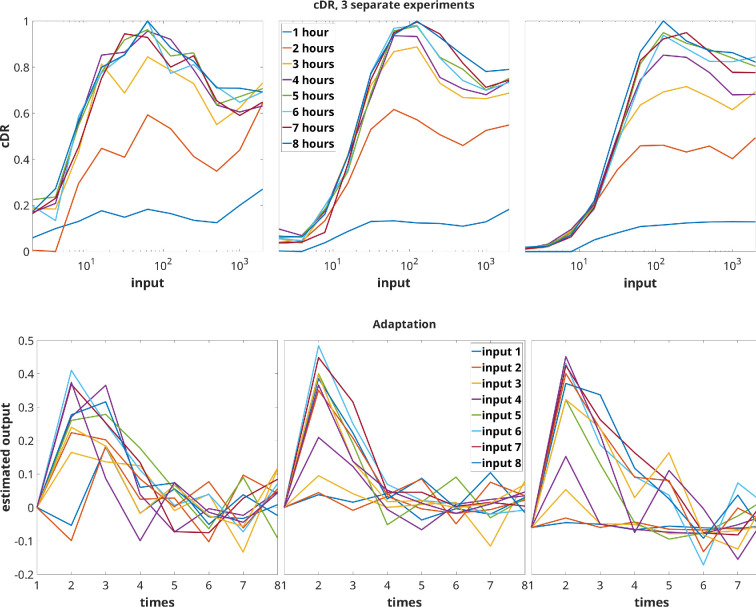
Top: cDR plots of individual experiments, and measured at different times. Bottom: adaptation behaviour in individual experiments. Output y(t) is estimated from individual cumulative z(t) plots in respective top panels.

Back to the experimental data, one may ask whether the T-cell experiments point to adaptation, that is, if $y_{u}(t)$ is independent of u for large t. Since only $z(t)=\int_{0}^{t}y(s)\;ds$ is experimentally available, we need to estimate the output y(t) by taking time-derivatives of z(t). To obtain a more meaningful estimate than would be obtained from the averages shown in figure 3, we consider instead the separate plots of z(t) from each experiment. The top panel in figure 6 shows again the cDRs for various times (t=1 to 8 hours), but now with separate plots from each experiment, starting from the data that were used to generate fig. 1 in the SI of [6].

Using first-order differences, and imposing a zero value at the start of the experiment: y(0)=0, we then derived estimates of y(t) for the various input values and the three experimental replicates shown in figure 6. See the bottom panel of figure 6. These estimates are very rough, because the time steps are large (1 hour), and in any event experimental data is subject to noise. Nonetheless, these data strongly suggests that adaptation, in the sense that the output y(t) approaches as time increases a value (in this case y=0), which is the same no matter what the input (drug dose). The estimated negative values of y(t) at certain time points are likely due to numerical errors or to the fact that there is some cytokine present in the experimental wells which does not arise from the stimulation, so that the values shown are relative to this baseline. In addition to adaptation, the data strongly suggests that the system, or at least its integrated output, is *scale-invariant* (performs ‘fold change detection’) in the sense of [7,8], at least for large enough input values: the transient outputs are roughly the same (for inputs 3−8), which is a property of systems IFFL2 and the IFB system as discussed below.

### 1.3. Review: network motifs for adaptation

As discussed (‘perfect’) adaptation to constant inputs means that, no matter the value of an input, the value of $y_{u}(t)$ for large t will be the same. Generally speaking, adaptation requires one of two mechanisms for adaptation: incoherent feedforward or negative feedback [9–11].

*IFFLs* are a type of network motifs that are capable of adaptation [12,13]. In an IFFL, the input u induces formation of the reporter y but also acts as a delayed inhibitor, through one or more intermediary control variables. Feedforward motifs are statistically overrepresented in biological systems from bacterial to mammalian cells [14,15]. IFFL’s have been argued to underlie mechanisms involved in such varied contexts as microRNA-mediated loops [16], MAPK pathways [17,18], insulin release [19,20], intracellular calcium release [21,22], *Dictyostelium* and neutrophil chemotaxis [23,24], NF-κB activation [25], and microRNA regulation [26], as well as metabolic regulation of bacterial carbohydrate uptake and other substrates [27,28]. IFFL’s may also play a role in immunology, enabling the recognition of dynamic changes in antigen presentation [29] and have been used in synthetic biology to control protein expression under DNA copy variability [30,31]. The paper [32] provided a large number of additional references, and carried out a computational exploration of IFFLs that lead to non-monotonic DRs and/or adaptation.

In *IFB* loops, the intermediate variable or variables provide a type of memory that integrates the ‘error’ between y(t) and a steady-state value $y_{0}$. IFB’s arise in biological systems ranging from *E. coli* chemotaxis [33] and regulation of tryptophan [34] to human physiological control such as blood calcium homeostasis [35] or neuronal control of the prefrontal cortex [36] to synthetic circuits for adaptation [37–39]. We remark that IFB is in a sense universal for adaptation, because nonlinear changes of coordinates can recast IFFLs that adapt into integral feedback form [40], but these coordinate changes may have no physical interpretation and hence lack interpretability. Moreover, IFBs are known to provide extra degrees of robustness to the adaptation property because, unlike the IFFL, the underlying mechanism can sense and correct for perturbations to the output variable y(t). This theme is explored in greater detail in recent papers, where it is shown that IFBs arise naturally when one considers adapting circuits that exhibit a *maximal* form of robustness [41] or robustness that is independent of the reaction kinetics [42]. The results in these papers apply to arbitrarily-sized networks, and they explicitly identify the network species that create the IFB required for adaptation.

Three paradigmatic circuits with two variables, two types of incoherent loops and one type of IFB, have been much studied, in particular in the context of the ‘fold change’ or ‘scale-invariance’ property in biology [7,8,43], and these systems, discussed next, are the focus of our study (Observe that nonlinearity is essential for non-monotonic cDR’s to be possible, since for linear systems, such as the IFFL $\dot{x}=-x+u$, $\dot{y}=x-u-y$ or the IFB $\dot{x}=y$, $\dot{y}=-x-y+u$, DRs and cDRs will be linear in u.) Although the concepts introduced here are broadly applicable, we focus our work in these three motifs, but we expect that our results will encourage further work in the direction of characterizing cDR properties for more complex systems.

### 1.4. Three paradigmatic examples of adapting systems

**Figure 7.**
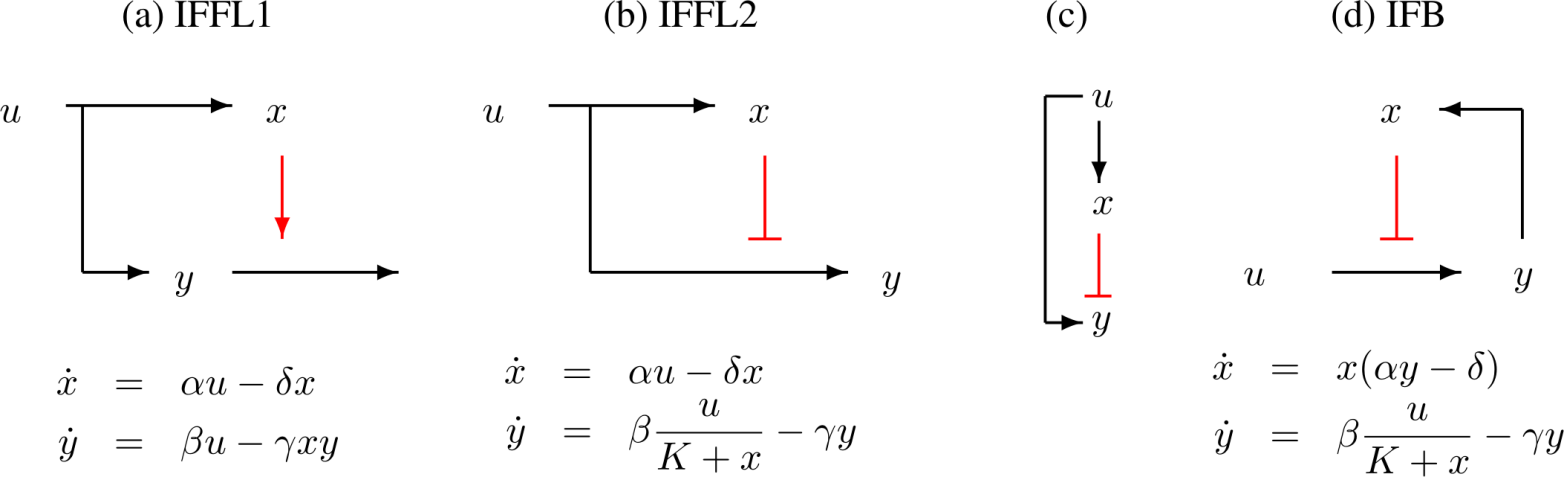
Three examples of systems: (a) is an IFFL with degradation enhancement, (b) is an IFFL with production inhibition and (d) is an IFB system. The input u is assumed to be a positive constant, and x, y are abundances of a quantity of interest, such as concentration of a protein or mRNA. Arrows ‘→’ indicate positive effect (activation) and blunt edges ‘⊣’ denote negative effects (inhibition). Both (a) and (b) have the same qualitative relation, schematically represented by diagram in (c), between activation of x and y by the input u, and inhibition of y by x, but their dynamical properties are very different. Dynamics can be described by pairs of differential equations for the abundances x(t) and y(t) as a function of time, as shown below the diagrams. State variables x(t) and y(t) are taken to be non-negative. When K=0, it is assumed that x(t)>0 for all t. In these equations, α, β, γ, δ and K are all positive constants.

In this article, we will consider three types of two-species systems, shown schematically in figure 7. These are the systems studied in [7,8]. In all these examples, u refers to a positive constant input, x is the concentration of a ‘controller’ species and the output variable y is the concentration of the ‘regulated’ or output species.

### 1.5. Steady states and perfect adaptation

Let us compute the steady states, obtained by setting the right-hand sides of the differential equations to zero, for the systems shown in figure 7. For the IFFL1 system (a) we have:


$$\bar{x}=\frac{\alpha }{\delta }u\;,\;\bar{y}=\frac{\beta }{\gamma \bar{x}}u=\frac{\beta }{\gamma }\frac{\delta }{\alpha u}u=\frac{\beta \delta }{\gamma \alpha }\;.$$

$$\bar{x}=\frac{\alpha }{\delta }u,\;\bar{y}=\frac{\beta }{\gamma (K+\bar{x})}u=\frac{\beta }{\gamma }\frac{\delta }{\left(\delta K+\alpha u\right)}u=\frac{\beta \delta }{\gamma \alpha }\text{ when }K=0.$$

Finally, for the IFB system (c) we have that there are two types of steady states:


$$(\bar{x},\bar{y})\;=\;\left(0,\frac{\beta u}{\gamma K}\right)$$

and, for non-zero $\bar{x}$ and assuming $K<\frac{\beta \delta }{\gamma \alpha }u$ (for example, if K=0):


$$(\bar{x},\bar{y})\;=\;\left(\frac{\beta \alpha }{\delta \gamma }u-K,\frac{\delta }{\alpha }\right).$$

In all three cases, if K=0, the system is perfectly adapting.

### 1.6. Scale invariance

In addition, when K=0 both systems IFFL2 and IFB have the scale-invariance (or ‘fold-change detection’, FCD) property [7,8]. This means that the output variable y(t) satisfies the same differential equation, independently of rescalings of the input u by any constant factor p, as shown by the following simple calculation:


$$\begin{array}{rlrl} \dot{x} & =\alpha u-\delta x & \dot{x} & =\alpha xy-\delta x\\ \dot{y} & =\beta \frac{u}{x}-\gamma y & \dot{y} & =\beta \frac{u}{x}-\gamma y\\ \Rightarrow \dot{\left(px\right)} & =\alpha (pu)-\delta (px) & \Rightarrow \dot{\left(px\right)} & =\alpha (px)y-\delta (px)\\ \dot{y} & =\beta \frac{\mathrm{p̸}u}{\mathrm{p̸}x}-\gamma y & \dot{y} & =\beta \frac{\mathrm{p̸}u}{\mathrm{p̸}x}-\gamma y. \end{array}$$

System IFFL1, in contrast, admits no such symmetries.

### 1.7. Stability

For both systems IFFL1 (a) and IFFL2 (b) in figure 7, the respective steady states $\left(\bar{x},\bar{y}\right)$ are globally asymptotically stable with respect to initial conditions in the positive quadrant x>0, y>0. This is very simple to show. The variable x(t) is the solution of a one-dimensional stable linear system, hence converges exponentially to $\frac{\alpha }{\delta }u$. The variable y is the solution of a time-dependent linear system, with a constant negative rate −γ for IFFL2, and a rate for IFFL1 which converges to the strictly negative number $-\frac{\alpha \gamma }{\delta }u$, and hence also exponentially converges to its steady-state value (see [13] for details, as well as similar results for other IFFL configurations).

The proof of stability for the feedback system IFB (d) in figure 7 requires more work. We proceed by extending the proof from [8], which covered only the case K=0. We will assume that $K<\frac{\beta \delta }{\gamma \alpha }u$, which holds in particular if K=0. We want to global asymptotic stability with respect to initial conditions with x(0) positive and y(0) non-negative (or even arbitrary), when u is a positive constant, for the two-dimensional system IFB evolving on ${\mathbb{R}}_{>0}\times \mathbb{R}$ with equations $\dot{x}=x(\alpha y-\delta )$, $\dot{y}=\beta \frac{u}{K+x}-\gamma y$.

It is convenient to change coordinates $\tilde{x}:=\ln\;x$ and $\tilde{y}=\alpha y-\delta$, so that we reduce to the study of the system in ${\mathbb{R}}^{2}$ with equations (dropping tildes):


$$\begin{array}{rl} \dot{x} & =y\\ \dot{y} & =\frac{\alpha \beta u}{K+{\text{e}}^{x}}-\gamma (y+\delta ) \end{array}$$

and we wish to prove the global asymptotic stability of the unique steady state


$$\bar{x}=\ln\left(\frac{\alpha \beta u}{\gamma \delta }-K\right),\;\bar{y}=0\;.$$

Introducing $c:=\gamma \delta =\frac{\alpha \beta u}{K+e^{\bar{x}}}$, $f(x)=-\frac{\alpha \beta u}{K+e^{x}}+c$, and k(y)=γ(y+δ)−c=γy, we can write our system as


$$\begin{array}{rl} \dot{x} & =y\\ \dot{y} & =-f(x)-k(y) \end{array}$$

with $f(\bar{x})=0$ and $k(\bar{y})=0$. In other words, we have a mass–spring system $\ddot{x}+k(\dot{x})+f(x)=0$ with nonlinear damping $k(\dot{x})$ and nonlinear spring constant f(x). This suggests the use of energy as a Lyapunov function. The map f is strictly increasing, positive for $x>\bar{x}$ and negative otherwise, and similarly, for k with respect to $\bar{y}=0$. Let us define


$$V(x,y)\;:=\;\int_{\bar{x}}^{x}f(r)\;dr\;+\;\frac{y^{2}}{2}\;.$$

By definition, $V(\bar{x},0)=0$ and V(x,y)>0 for all $(x,y)≠\left(\bar{x},0\right)$. We also have that $\frac{\partial^{2}V}{\partial^{2}x}=f^{\prime }(x)>0$, and $\frac{\partial^{2}V}{\partial^{2}y}=1>0$, (and mixed partial derivatives are zero), V is a strictly convex and thus a proper (also called radially unbounded or coercive) function, thus a Lyapunov function candidate. The derivative of V along trajectories is


$$f(x)\;y\;+\;y\left[-f(x)-k(y)\right]\;=\;-\gamma y^{2}\;\leq \;0$$

for all (x,y), and if this derivative only vanishes identically along a trajectory, then y(t)≡0, which in turn implies, when substituted into $0=\dot{y}=-f(x)-0$ that f(x(t))≡0, i.e. that $x(t)\equiv \bar{x}$. The LaSalle invariance principle (e.g. [44]) then allows us to conclude global asymptotic stability.

### 1.8. Outline of paper

Denoting the input-dependent dynamics as $\left(x_{u}(t),y_{u}(t)\right)$ for t≥0, we define the DR and the cDR at time T as


$$\mathrm{DR}(u,T)=y_{u}(T)\;\text{ and }\;\mathrm{cDR}(u,T)=\int_{0}^{T}y_{u}(t)dt.$$

In each example, our aim is to determine whether the mapping u↦cDR(u,T) is monotonically increasing. If this monotonicity does not hold universally, we seek to identify sufficient conditions under which it does. It is straightforward to note that if the map u↦DR(u,T) is monotonically increasing, then the same holds for the map u↦cDR(u,T). Observe that if the system is linear, both DR(u,T) and cDR(u,T) will be linear (and therefore monotonic) functions of *u*. Hence, non-monotonicity is exclusively a property of nonlinear systems.

Let us review the specific examples that we consider. The first is the IFFL1 system shown in figure 7*a* with equations given by


(1.5)x˙=αu−δx(1.6)y˙=βu−γxy,

with initial state $x(0)=x_{0}$ and $y(0)=\frac{\beta \delta }{\alpha \gamma }$. The initial state for x is arbitrary non-negative, while the initial state for y is its steady-state value which is independent of u>0.

The second example is the IFFL2 system (see figure 7*b*) given by


(1.7)x˙=αu−δx(1.8)y˙=βuK+x−γy,

where the initial states $x_{u}(0)=x_{0}>0$ and $y_{u}(0)=y_{0}>0$ are arbitrary.

As the final example we have the IFB system (see figure 7*c*) given by


(1.9)x˙=x(αy−δ)(1.10)y˙=βuK+x−γy

with initial states $x(0)=x_{0}>0$ (arbitrary) and $y(0)=\frac{\delta }{\alpha }$, which is the steady state for y which is independent of u.

We remark that for all of these examples, u↦DR(u,T) (and therefore also u↦cDR(u,T)) is monotonically increasing for *small* T. This is because $\partial_{u}{\dot{y}}_{u}(0)=\beta$ for IFFL1, and $\partial_{u}{\dot{y}}_{u}(0)=\beta /\left(K+x_{0}\right)$ for IFFL2 and IFB. From $y_{u}(t)=y_{0}+{\dot{y}}_{u}(0)t+o(t)$, it follows that $\partial_{u}y_{u}(T)\approx \beta T$ and $\partial_{u}y_{u}(T)\approx \left(\beta T\right)/\left(K+x_{0}\right)$, respectively, and both are positive. The situation is far less trivial for larger times T.

The rest of this article is organized as follows. In the rest of this introduction, we review some other areas of application of cDR’s. In §2, we prove that for IFFL1, even though the map u↦DR(u,T) may not be monotonic, the map u↦cDR(u,T) is always monotonically increasing, irrespective of the values of T, $x_{0}$, δ and γ. In §3, we show that the situation is much simpler for IFFL2, in the sense that both DR(u,T) and cDR(u,T) are monotonically increasing functions of u, regardless of the choice of T, initial states, and the model parameters. Finally, in §4, we show that for the IFB system the map u↦cDR(u,T) is not monotonic in general and find a sufficient condition under which monotonicity holds.

### 1.9. Further motivations for the study of cumulative dose responses

In pharmacology and biomedical research, measuring the cumulative amount or ‘area under the curve’ (AUC) of the concentration or abundance of a substance (such as an antibody, cytokine, hormone or metabolite) secreted over a time period, which we termed the cDR, is essential for understanding drug efficacy, toxicity and biological responses. To illustrate the potential wide interest of cDR theory, we briefly discuss here a few examples of such measurements.

*Cytokine release assays* are laboratory techniques that typically measure the cumulative secretion of cytokines, which are small signalling proteins produced by immune cells in response to stimuli such as drugs, pathogens or immune activators. Cytokines regulate immune and inflammatory responses as well as cell growth, differentiation, survival and tissue repair. Examples are pro-inflammatory (IL-6, IL-1β, TNF-α and IFN-γ) and anti-inflammatory (IL-10 and IL-4) cytokines, and growth factors (GM-CSF and VEGF). Typical techniques used for measuring cumulative release of cytokines are enzyme-linked immunosorbent assay (ELISA) and multiplex immunoassays such as Luminex${,}^{\text{ ®}}$. For example [45], describes means of measuring pro-inflammatory cytokines in the central nervous system as a way to monitor neuroinflammatory responses to trauma, infection and neurodegenerative diseases. That paper describes an *in vivo* immunosensing device in which an optical fibre is implanted for a period in the brain of a rodent to capture (by binding to a specific antibody) the cumulative release of a specific cytokine within a region of interest; ELISA is then conducted, in order to determine the cumulative amount of cytokine bound to the fibre. As another example, one of the most common adverse events associated with T-cell bispecific antibody therapies (which are themselves an interesting subject for mathematical modelling [46]) is cytokine release syndrome (CRS), whose symptoms include fever, hypotension, respiratory deficiency and possible multi-organ failure. The paper [47] highlighted the use of Luminex${,}^{\text{ ®}}$ and AUC plots of cytokine release to evaluate different therapeutic approaches to the mitigation of CRS.

The *metabolic clearance of drugs* is often assessed by measuring the cumulative amount of a metabolite over time in biological fluids. Typically, after a drug or prodrug is administered, plasma concentrations are measured as a function of time, and the area under the concentration–time curve is computed [48], thus providing critical insights into drug metabolism, pharmacokinetics and hepatic or renal clearance mechanisms. Hence, helping to understand drug efficacy and safety. The paper [49] provided a systematic evaluation of cumulative drug excretion in clinical pharmacokinetics, emphasizing its role in dosing regimens and safety evaluations. It described various measures of drug accumulation, including the AUC on a graph plotting plasma concentration against time.

As one last example, consider the routine *A1c blood test* (also known as glycated haemoglobin or HbA1c test), which is key to diabetes diagnosis and management. A1c measures *average* blood glucose levels over a period of about 2−3 months before testing, and one could therefore think of it as being proportional to the integral of glucose concentration over that period. The integration effect, compared with measuring of short-term fluctuations in blood glucose, is due to the long lifespan of red blood cells, whose haemoglobin binds glucose to form HbA1c.

## 2. Unconditional monotonicity of the cumulative dose response for incoherent feedforward loop 1

We shall prove the monotonicity of the map u↦cDR(u,T) in multiple steps. As the first step, we simplify the system (1.5) and (1.6) in §2.1 to obtain a parameter-free form that is more amenable to analysis. In §2.2, we then derive explicit expressions for the cDR and its partial derivative with respect to u. This partial derivative is given by an integral expression, and in §2.3, we establish a couple of properties of the integrand and the asymptotic value of the integral expression. These results enable us to prove the monotonicity of the cDR in §2.3 by demonstrating that its partial derivative with respect to the input u is always non-negative.

### 2.1. Simplifying the system

Let $\left(x_{u}(t),y_{u}(t)\right)$ be the solution of (1.5) and (1.6) for t≥0. We scale time by $\delta^{-1}$ and the state values by a suitable ratio to define


$$\begin{array}{r} \hat{x}(t)=\frac{\gamma }{\delta }x(\delta^{-1}t)\;\text{and}\;\hat{y}(t)=\frac{\gamma }{\delta }\frac{\alpha }{\beta }y(\delta^{-1}t). \end{array}$$

Then, the dynamics of this rescaled system are given by


$$\begin{array}{rl} \dot{\hat{x}}(t) & =\frac{\gamma }{\delta^{2}}\dot{x}(\delta^{-1}t)=\frac{\gamma }{\delta^{2}}\left(-\delta x(\delta^{-1}t)+\alpha u\right)=-\frac{\gamma }{\delta }x(\delta^{-1}t)+\frac{\gamma }{\delta^{2}}\alpha u\\ & =-\hat{x}(t)+\frac{\gamma }{\delta^{2}}\alpha u,\\ \dot{\hat{y}}(t) & =\frac{\gamma }{\delta^{2}}\frac{\alpha }{\beta }\dot{y}(\delta^{-1}t)=\frac{\gamma }{\delta^{2}}\frac{\alpha }{\beta }\left(-\gamma x(\delta^{-1}t)y(\delta^{-1}t)+\beta u\right)=-\frac{\gamma^{2}}{\delta^{2}}x(\delta^{-1}t)\frac{\alpha }{\beta }y(\delta^{-1}t)+\frac{\gamma }{\delta^{2}}\alpha u\\ & =-\hat{x}(t)\hat{y}(t)+\frac{\gamma }{\delta^{2}}\alpha u. \end{array}$$

Therefore, if we define


$$\hat{u}=\frac{\gamma }{\delta^{2}}\alpha u$$

then $\left(\hat{x}(t),\hat{y}(t)\right)$ satisfies the ODEs


$$\begin{array}{rl} \dot{\hat{x}}(t) & =-\hat{x}(t)+\hat{u}\\ \dot{\hat{y}}(t) & =-\hat{x}(t)\hat{y}(t)+\hat{u} \end{array}$$

with initial states $\hat{x}(0)=\frac{\gamma }{\delta }x_{0}$ and $\hat{y}(0)=1$. Let $\left({\hat{x}}_{\hat{u}}(t),{\hat{y}}_{\hat{u}}(t)\right)$ be the solution of this system. To prove the result it suffices to show that the map


$$\begin{array}{r} \hat{u}\mapsto \int_{0}^{T}{\hat{y}}_{\hat{u}}(t)dt \end{array}$$

is monotonically increasing for any T>0.

Henceforth, we shall drop the hats for notational convenience and suppose that the dynamics is given by


(2.1)x˙(t)=−x(t)+u(2.2)y˙(t)=−x(t)y(t)+u

with initial state $x(0)=x_{0}$ (arbitrary) and y(0)=1. Letting $\left(x_{u}(t),y_{u}(t)\right)$ be the solution of this system, we shall show that the cDR map


$$\begin{array}{r} u\mapsto \int_{0}^{T}y_{u}(t)dt \end{array}$$

is monotonically increasing for any T>0. In order to prove this we will prove that for any T and u


(2.3) $$\begin{array}{r} \int_{0}^{T}\partial_{u}y_{u}(t)dt\geq 0, \end{array}$$

where $\partial_{u}$ denotes the partial derivative with respect to u.

### 2.2. Derivation of explicit expressions

We now develop explicit expressions for $x_{u}(t)$, $y_{u}(t)$, $\int_{0}^{T}y_{u}(t)dt$ and $\int_{0}^{T}\partial_{u}y_{u}(t)dt$. It is easy to see that


(2.4) $$\begin{array}{r} x_{u}(t)=x_{0}e^{-t}+(1-e^{-t})u=u-(u-x_{0})e^{-t}, \end{array}$$

which also implies that


(2.5) $$\begin{array}{r} \partial_{u}x_{u}(t)=1-e^{-t}. \end{array}$$

Note that ODE (2.2) can be written as


$$\begin{array}{r} {\dot{y}}_{u}+x_{u}y_{u}=u. \end{array}$$

Multiplying with the integrating factor $e^{\int_{0}^{t}x_{u}(s)ds}$ on both sides we obtain


$$\begin{array}{r} \frac{d}{dt}e^{\int_{0}^{t}x_{u}(s)ds}y_{u}(t)=ue^{\int_{0}^{t}x_{u}(s)ds}. \end{array}$$

Integrating both sides and using $y_{u}(0)=1$ we get the usual variation of the parameters formula:


$$\begin{array}{r} y_{u}(t)=e^{-\int_{0}^{t}x_{u}(s)ds}+u\int_{0}^{t}e^{-\int_{s}^{t}x_{u}(w)dw}ds. \end{array}$$

From (2.1), we know that


$$\begin{array}{r} \int_{s}^{t}x_{u}(w)dw=u(t-s)-(x_{u}(t)-x_{u}(s))=u(t-s)-(u-x_{0})e^{-t}(e^{t-s}-1), \end{array}$$

where the second relation follows from (2.4). Plugging this in the previous expression for $y_{u}(t)$ we get


(2.6) $$\begin{array}{rl} y_{u}(t) & =e^{(u-x_{0})(1-e^{-t})-ut}+u\int_{0}^{t}e^{\left(u-x_{0})e^{-t}(e^{t-s}-1)-u(t-s\right)}ds\\ & =e^{(u-x_{0})(1-e^{-t})-ut}+u\int_{0}^{t}e^{(u-x_{0})e^{-t}(e^{s}-1)-us}ds, \end{array}$$

where to derive the last expression we have made a change of variable from (t−s) to s.

Note that by changing the order to integration in the second term in the r.h.s. below we obtain


$$\begin{array}{rl} \int_{0}^{T}y_{u}(t)dt & =\int_{0}^{T}e^{(u-x_{0})(1-e^{-t})-ut}dt+u\int_{0}^{T}\int_{0}^{t}e^{(u-x_{0})e^{-t}(e^{s}-1)-us}dsdt\\ & =\int_{0}^{T}e^{(u-x_{0})(1-e^{-t})-ut}dt+u\int_{0}^{T}e^{-us}\left(\int_{s}^{T}e^{\left(u-x_{0})e^{-t}(e^{s}-1\right)}dt\right)ds. \end{array}$$

Making the change of variable $t\mapsto \left(u-x_{0})e^{-t}(e^{s}-1\right)$ we see that


$$\begin{array}{rl} \int_{s}^{T}e^{\left(u-x_{0})e^{-t}(e^{s}-1\right)}dt & =\int_{\left(u-x_{0})(e^{s-T}-e^{-T}\right)}^{\left(u-x_{0})(1-e^{-s}\right)}\frac{e^{t}}{t}dt\\ & =\text{Ei}\left(\left(u-x_{0})(1-e^{-s}\right)\right)-\text{Ei}\left(\left(u-x_{0})(e^{s-T}-e^{-T}\right)\right), \end{array}$$

where Ei(t) is the special *exponential integral* function defined as


(2.7) $$\begin{array}{r} \text{Ei}(t)=\int_{-\infty }^{t}\frac{e^{w}}{w}dw. \end{array}$$

Plugging this in we obtain


(2.8) $$\begin{array}{r} \int_{0}^{T}y_{u}(t)dt=\int_{0}^{T}e^{(u-x_{0})(1-e^{-t})-ut}dt+u\int_{0}^{T}e^{-us}I(s,T)ds, \end{array}$$

where


$$I(s,T)=\text{Ei}\left((u-x_{0})(1-e^{-s})\right)-\text{Ei}\left((u-x_{0})(e^{s-T}-e^{-T})\right).$$

Observe that at $s\to T^{-}$ we have I(s,T)→I(T,T)=0 and we claim that as $s\to 0^{+}$, we have I(s,T)→T. To see this note that as $s\to 0^{+}$, both $\left(1-e^{-s}\right)$ and $\left(e^{s-T}-e^{-T}\right)$ approach 0 and close to 0 we have


$$\begin{array}{rl} & \text{Ei}\left(\left(u-x_{0})(1-e^{-s}\right)\right)-\text{Ei}\left(\left(u-x_{0})(e^{s-T}-e^{-T}\right)\right)\\ & =\int_{\left(u-x_{0})(e^{s-T}-e^{-T}\right)}^{\left(u-x_{0})(1-e^{-s}\right)}\frac{e^{t}}{t}dt\\ & \approx \int_{\left(u-x_{0})(e^{s-T}-e^{-T}\right)}^{\left(u-x_{0})(1-e^{-s}\right)}\frac{1}{t}dt\\ & =\log((u-x_{0})(1-e^{-s}))-\log((u-x_{0})(e^{s-T}-e^{-T}))\\ & =\log\left(\frac{\left(u-x_{0})(1-e^{-s}\right)}{\left(u-x_{0})e^{s-T}(1-e^{-s}\right)}\right)\\ & =T-s, \end{array}$$

which is just T as s→0+. By the definition of the Ei function (2.7)


$$\begin{array}{r} \frac{d}{ds}\text{Ei}(s)=\frac{e^{s}}{s} \end{array}$$

and hence by the chain rule


$$\begin{array}{rl} \frac{d}{ds}\text{Ei}\left((u-x_{0})(1-e^{-s})\right) & =\frac{e^{\left(u-x_{0})(1-e^{-s}\right)}}{\left(u-x_{0})(1-e^{-s}\right)}(u-x_{0})e^{-s}=e^{\left(u-x_{0})(1-e^{-s}\right)}\frac{e^{-s}}{1-e^{-s}} \end{array}$$

and


$$\begin{array}{rl} \frac{d}{ds}\text{Ei}\left((u-x_{0})(e^{s-T}-e^{-T})\right) & =\frac{e^{\left(u-x_{0})(e^{s-T}-e^{-T}\right)}}{\left(u-x_{0})(e^{s-T}-e^{-T}\right)}(u-x_{0})e^{s-T}\\ & =e^{\left(u-x_{0})(e^{s-T}-e^{-T}\right)}\frac{e^{s-T}}{e^{s-T}-e^{-T}}\\ & =e^{\left(u-x_{0})(e^{s-T}-e^{-T}\right)}\frac{1}{1-e^{-s}}. \end{array}$$

Using these expressions we can write the derivative of I(s,T) as


$$\begin{array}{rl} \frac{d}{ds}I(s,T) & =\frac{d}{ds}\text{Ei}\left((u-x_{0})(1-e^{-s})\right)-\frac{d}{ds}\text{Ei}\left((u-x_{0})(e^{s-T}-e^{-T})\right)\\ & =e^{\left(u-x_{0})(1-e^{-s}\right)}\frac{e^{-s}}{1-e^{-s}}-e^{\left(u-x_{0})(e^{s-T}-e^{-T}\right)}\frac{1}{1-e^{-s}}. \end{array}$$

Therefore, applying integration by parts to the second term in the r.h.s of (2.8) we get


$$\begin{array}{rl} & u\int_{0}^{T}e^{-us}I(s,T)ds=\lim_{s\to 0^{+}}\;I(s,T)-\lim_{s\to T^{-}}\;I(s,T)+\int_{0}^{T}e^{-us}\frac{d}{ds}I(s,T)ds\\ & =T+\int_{0}^{T}e^{-us}\left(e^{\left(u-x_{0})(1-e^{-s}\right)}\frac{e^{-s}}{1-e^{-s}}-e^{\left(u-x_{0})(e^{s-T}-e^{-T}\right)}\frac{1}{1-e^{-s}}\right)ds. \end{array}$$

Upon substituting this term in the r.h.s. of (2.8) we obtain


(2.9) $$\begin{array}{rl} \int_{0}^{T}y_{u}(t)dt & =\int_{0}^{T}e^{(u-x_{0})(1-e^{-t})-ut}dt+T\\ & +\int_{0}^{T}e^{-us}\left(e^{\left(u-x_{0})(1-e^{-s}\right)}\frac{e^{-s}}{1-e^{-s}}-e^{\left(u-x_{0})(e^{s-T}-e^{-T}\right)}\frac{1}{1-e^{-s}}\right)ds. \end{array}$$

Note that by rearranging and simplifying, (2.9) can be expressed as:


(2.10) $$\begin{array}{rl} \int_{0}^{T}y_{u}(t)dt & =T+\int_{0}^{T}\frac{e^{-us}}{1-e^{-s}}\left(e^{\left(u-x_{0})(1-e^{-s}\right)}-e^{\left(u-x_{0})(e^{s-T}-e^{-T}\right)}\right)ds. \end{array}$$

Differentiating (2.10) with respect to u we obtain


$$\begin{array}{r} \int_{0}^{T}\partial_{u}y_{u}(t)dt=-\int_{0}^{T}s\frac{e^{-us}}{1-e^{-s}}\left(e^{\left(u-x_{0})(1-e^{-s}\right)}-e^{\left(u-x_{0})(e^{s-T}-e^{-T}\right)}\right)ds\\ +\int_{0}^{T}e^{-us}\left(e^{\left(u-x_{0})(1-e^{-s}\right)}-e^{\left(u-x_{0})(e^{s-T}-e^{-T}\right)}e^{s-T}\right)ds \end{array}$$

which can be rewritten as


(2.11) $$\begin{array}{rl} & \int_{0}^{T}\partial_{u}y_{u}(t)dt=\int_{0}^{T}e^{-us}(e^{\left(u-x_{0})(1-e^{-s}\right)}\left(1-\frac{s}{1-e^{-s}}\right)\\ & +e^{\left(u-x_{0})(e^{s-T}-e^{-T}\right)}\left(\frac{s}{1-e^{-s}}-e^{s-T}\right))ds. \end{array}$$

### 2.3. Two useful results

We now establish a couple of useful results that will help us in proving the monotonicity property of the cDR.

**Lemma 2.1.** *Fix any positive* T *and* κ, *and for* s∈[0,T] *define functions*


$$\begin{array}{r} \rho (s)=(1-e^{-s})(1-e^{s-T}) \end{array}$$

*and*


(2.12) $$\begin{array}{r} f_{\kappa }(s)=e^{-\kappa \rho (s)}\left(\frac{s}{1-e^{-s}}-e^{s-T}\right)-\left(\frac{s}{1-e^{-s}}-1\right). \end{array}$$

*Then, the function* $f_{\kappa }(s)$ *can cross the* x*-axis only once in the interval* [0,T].

*Proof*. Note that $\rho (s)=(1-e^{-s})(1-e^{s-T})=1+e^{-T}-e^{-s}-e^{s-T}$. Setting $f_{\kappa }(s)=0$ we get


$$\begin{array}{r} s+e^{-s}-1=e^{-\kappa \rho (s)}(s-e^{s-T}+e^{-T})=e^{-\kappa \rho (s)}(s+e^{-s}-1+\rho (s)), \end{array}$$

$$\begin{array}{r} s+e^{-s}-1=\frac{\rho (s)}{e^{\kappa \rho (s)}-1}. \end{array}$$

Let $g_{\kappa }(s)$ be the function


$$\begin{array}{r} g_{\kappa }(s)=(s+e^{-s}-1)-\frac{\rho (s)}{e^{\kappa \rho (s)}-1}. \end{array}$$

To prove the lemma it suffices to show that the function $g_{\kappa }(s)$ is monotonically increasing, which we shall show by proving that $g_{\kappa }^{\prime }(s)\geq 0$ for all s∈[0,T]. For convenience let us define a function


$$\begin{array}{r} \phi (z)=\frac{z}{e^{z}-1}. \end{array}$$

Then, we can write $g_{\kappa }(s)$ as


$$\begin{array}{r} g_{\kappa }(s)=(s+e^{-s}-1)-\frac{1}{\kappa }\phi (\kappa \rho (s)) \end{array}$$

which shows that


$$\begin{array}{r} g_{\kappa }^{\prime }(s)=1-e^{-s}-\phi^{\prime }(\kappa \rho (s))\rho^{\prime }(s)=1-e^{-s}-\phi^{\prime }(\kappa \rho (s))(e^{-s}-e^{s-T}). \end{array}$$

Suppose we can show that


(2.13) $$\begin{array}{r} -1\leq \phi^{\prime }(z)\leq 0\;\text{for all}\;z\geq 0. \end{array}$$

Then, for $s\in \left[0,\frac{T}{2}\right]$ we have $(e^{-s}-e^{s-T})>0$ and so $g_{\kappa }^{\prime }(s)\geq 1-e^{-s}\geq 0$. On the other hand, for $s\in \left[\frac{T}{2},T\right]$, $(e^{-s}-e^{s-T})<0$ and using $\phi^{\prime }(z)\geq -1$, we obtain


$$g_{\kappa }^{\prime }(s)\geq 1-e^{-s}+(e^{-s}-e^{s-T})=1-e^{s-T}\geq 0.$$

Hence, to prove the lemma we just need to prove the inequality (2.13) to which we come to now. Note that


$$\begin{array}{r} \phi^{\prime }(z)=\frac{1}{e^{z}-1}-\frac{ze^{z}}{(e^{z}-1{)}^{2}}=\frac{e^{z}-1-ze^{z}}{(e^{z}-1{)}^{2}}=-\frac{e^{z}(z+e^{-z}-1)}{(e^{z}-1{)}^{2}}. \end{array}$$

Since $z+e^{-z}-1\geq 0$ for any z≥0, we would have that $\phi^{\prime }(z)\leq 0$. Since the function ϕ(z) is monotonically decreasing for z≥0 we must have


(2.14) $$\begin{array}{lrr} & \underset{z>0}{\sup}\;\phi (z)=\lim_{z\to 0^{+}}\;\phi (z)=1. & \end{array}$$

Observe that we can write $\phi^{\prime }(z)$ as


$$\begin{array}{r} \phi^{\prime }(z)=\frac{e^{z}-1-z}{(e^{z}-1{)}^{2}}-\phi (z). \end{array}$$

As the first term is always positive, we have $\phi^{\prime }(z)\geq -\phi (z)$, which along with (2.14) shows that


$$\begin{array}{r} \underset{z>0}{\inf}\;\phi^{\prime }(z)\geq -\underset{z>0}{\sup}\;\phi (z)=-1. \end{array}$$

This completes the proof of the inequality (2.13) and concludes the proof of this lemma.∎

**Proposition 2.2.** *For any* u>0, *the integral*


(2.15) $$\begin{array}{lrr} & \int_{0}^{\infty }e^{-us}\left(\frac{s}{1-e^{-s}}-e^{u(1-e^{-s})}\left(\frac{s}{1-e^{-s}}-1\right)\right)ds & \end{array}$$

*has a positive value.*

*Proof*. Define a function


$$w(s)=s+e^{-s}-1.$$

Note that the integral (2.15) can be rewritten as


$$\begin{array}{r} I(u)=\int_{0}^{\infty }\left(\frac{se^{-us}-w(s)e^{-uw(s)}}{1-e^{-s}}\right)ds=\frac{d}{du}G(u), \end{array}$$

where


$$\begin{array}{r} G(u)=\int_{0}^{\infty }\left(\frac{e^{-uw(s)}-e^{-us}}{1-e^{-s}}\right)ds. \end{array}$$

Hence, in order to prove that I(u) is positive, we just need to prove that the function G(u) is monotonically increasing. This is what we show next.

Using the fact that


$$\frac{e^{x}-1}{x}=\sum_{n=0}^{\infty }\frac{x^{n}}{(n+1)!},$$

we obtain


$$\begin{array}{rl} G(u) & =\int_{0}^{\infty }e^{-us}\left(\frac{e^{u(1-e^{-s})}-1}{1-e^{-s}}\right)ds\\ & =\sum_{n=0}^{\infty }\frac{u^{n+1}}{(n+1)!}\int_{0}^{\infty }e^{-us}(1-e^{-s}{)}^{n}ds. \end{array}$$

Applying the change of variable $t=1-e^{-s}$, we get


$$\begin{array}{rl} G(u) & =\sum_{n=0}^{\infty }\frac{u^{n+1}}{(n+1)!}\int_{0}^{1}(1-t{)}^{u-1}t^{n}dt. \end{array}$$

The integral on the right can be expressed in terms of the Gamma function Γ(x) as


$$\int_{0}^{1}(1-t{)}^{u-1}t^{n}dt=\frac{\Gamma (n+1)\Gamma (u)}{\Gamma (n+1+u)}=\frac{n!\Gamma (u)}{\Gamma (n+1+u)}.$$

Substituting this we obtain


$$\begin{array}{rl} G(u) & =\sum_{n=0}^{\infty }\frac{u^{n+1}}{\left(n+1\right)}\frac{\Gamma (u)}{\Gamma (n+1+u)}. \end{array}$$

Since Γ(x+1)=xΓ(x) we can express G(u) as


$$\begin{array}{rl} G(u) & =\sum_{n=0}^{\infty }\frac{1}{\left(n+1\right)}L_{n}(u), \end{array}$$

where


$$L_{n}(u)=\prod_{j=1}^{n}\left(\frac{u}{u+j}\right).$$

As each $L_{n}(u)$ is a product of positive monotonically increasing functions, the function G(u) is also monotonically increasing. This completes the proof of this proposition.∎

### 2.4. Proving monotonicity of the cumulative dose response

Finally, we now prove the monotonicity of the cDR by proving (2.3). For this we shall use the integral expression (2.11).

Let us first deal with the case $u\leq x_{0}$. In this scenario, $(u-x_{0})\leq 0$ and since $e^{s-T}\leq 1$ we have


$$e^{\left(u-x_{0})(e^{s-T}-e^{-T}\right)}=e^{\left(u-x_{0})e^{s-T}(1-e^{-s}\right)}\geq e^{\left(u-x_{0})(1-e^{-s}\right)}.$$

Therefore, the integrand on the r.h.s. of (2.11) can be bounded below


$$\begin{array}{rl} & e^{\left(u-x_{0})(1-e^{-s}\right)}\left(1-\frac{s}{1-e^{-s}}\right)+e^{\left(u-x_{0})(e^{s-T}-e^{-T}\right)}\left(\frac{s}{1-e^{-s}}-e^{s-T}\right)\\ & \geq e^{\left(u-x_{0})(1-e^{-s}\right)}\left(1-e^{s-T}\right)\geq 0. \end{array}$$

Hence, the integrand in (2.11) is always non-negative which establishes the monotonicity of cDR for $u\leq x_{0}$.

We now consider the case $u>x_{0}$. Note that in this case $x_{u}(t)$ (see (2.4)) is monotonically increasing from $x_{0}$ to u. Hence, $y_{u}(0)=1$ and


$$\begin{array}{r} {\dot{y}}_{u}(t)=-x_{u}(t)y_{u}(t)+u\geq -uy_{u}(t)+u, \end{array}$$

which allows us to conclude that $y_{u}(t)\geq 1$ for all t≥0, by a simple comparison argument. Suppose that $x_{0}\leq 1$. In this case, we prove that for any positive u, the trajectory $t\mapsto \partial_{u}y_{u}(t)$ can only change its sign once, to go from positive to negative, and then it will stay negative and asymptotically approach 0. To see this define


$$z_{u}(t)=(1-e^{-t})y_{u}(t)-1$$

and then


$$\begin{array}{rl} {\dot{z}}_{u}(t) & =e^{-t}y_{u}(t)+(1-e^{-t}){\dot{y}}_{u}(t)\\ & =e^{-t}y_{u}(t)+(1-e^{-t})(-x_{u}(t)y_{u}(t)+u)\\ & =e^{-t}y_{u}(t)+(1-e^{-t})u-x_{u}(t)(1-e^{-t})y_{u}(t)\\ & =e^{-t}y_{u}(t)+(1-e^{-t})u-x_{u}(t)-x_{u}(t)z_{u}(t)\\ & =e^{-t}(y_{u}(t)-x_{0})-x_{u}(t)z_{u}(t). \end{array}$$

Since we have assumed $x_{0}\leq 1$ we have the differential inequality


$$\begin{array}{r} {\dot{z}}_{u}(t)\geq -x_{u}(t)z_{u}(t). \end{array}$$

Hence, by the comparison argument, if there exists a $t_{1}$ such that $z_{u}(t_{1})\geq 0$, then for all $t\geq t_{1}$ we have $z_{u}(t)\geq 0$ which also implies that


$$y_{u}(t)\geq \frac{1}{1-e^{-t}}.$$

Now let $t_{1}$ be the first zero of the trajectory $\partial_{u}y_{u}(t)$. Observe that


$$\begin{array}{rl} \partial_{u}\dot{y_{u}}(t) & =-y_{u}(t)\partial_{u}x_{u}(t)-x_{u}(t)\partial_{u}y_{u}(t)+1\\ & =-(1-e^{-t})y_{u}(t)-x_{u}(t)\partial_{u}y_{u}(t)+1\\ & =-z_{u}(t)-x_{u}(t)\partial_{u}y_{u}(t). \end{array}$$

As $t_{1}$ is the first zero of the trajectory $\partial_{u}y_{u}(t)$ we have $\partial_{u}y_{u}(t)>0$ for $t<t_{1}$, and $\partial_{u}\dot{y_{u}}(t_{1})\leq 0$ and $\partial_{u}y_{u}(t_{1})=0$. Hence, we must have $z_{u}(t_{1})\geq 0$ and by previous arguments $z_{u}(t)\geq 0$ for all $t\geq t_{1}$. Therefore, in this interval $t\in \left(t_{1},\infty \right)$, we have the inequality


$$\partial_{u}\dot{y_{u}}(t)\leq -x_{u}(t)\partial_{u}y_{u}(t)$$

and since $\partial_{u}y_{u}(t_{1})=0$, the comparison argument shows that $\partial_{u}y_{u}(t)\leq 0$ for all $t\geq t_{1}$. Hence, the trajectory $t\mapsto \partial_{u}y_{u}(t)$ can only change its sign once, to go from positive to negative. Therefore, in order to prove $\int_{0}^{T}\partial_{u}y_{u}(t)dt\geq 0$ for any T, it suffices to prove that this holds in the limit T→∞. Letting T→∞ in (2.11) we arrive at


$$\begin{array}{r} \int_{0}^{\infty }\partial_{u}y_{u}(t)dt=\int_{0}^{\infty }e^{-us}\left(\frac{s}{1-e^{-s}}-e^{\left(u-x_{0})(1-e^{-s}\right)}\left(\frac{s}{1-e^{-s}}-1\right)\right)ds. \end{array}$$

Since $\frac{s}{1-e^{-s}}\geq 1$ for all s>0, in order to prove the positivity of this integral it suffices to prove its positivity for $x_{0}=0$, i.e.


(2.16) $$\begin{array}{lrr} & \int_{0}^{\infty }\partial_{u}y_{u}(t)dt=\int_{0}^{\infty }e^{-us}\left(\frac{s}{1-e^{-s}}-e^{u(1-e^{-s})}\left(\frac{s}{1-e^{-s}}-1\right)\right)ds>0, & \end{array}$$

which holds due to Proposition 2.2.

We now come to the case where $1<x_{0}<u$. Set $\kappa =\left(u-x_{0}\right)$ and let ρ(s) and $f_{\kappa }(s)$ be the functions defined in the statement of Lemma 2.1. Note that (2.11) can be written in terms of this function as


(2.17) $$\begin{array}{lrr} & \int_{0}^{T}\partial_{u}y_{u}(t)dt=\int_{0}^{T}e^{-us}e^{\kappa (1-e^{-s})}f_{\kappa }(s)ds. & \end{array}$$

Moreover


$$\begin{array}{r} \lim_{s\to 0^{+}}\;f_{\kappa }(s)=1-e^{-T}>0\;\text{and}\;f_{\kappa }(T)=0. \end{array}$$

Lemma 2.1 proves that the function $f_{\kappa }(s)$ can only cross the x-axis at most once in the interval [0,T]. If it does cross then it goes from positive to negative and it stays negative untill it becomes 0 at s=T. The same holds for the function $e^{-\kappa s}e^{\kappa (1-e^{-s})}f_{\kappa }(s)$. Hence, if we can prove that


(2.18) $$\begin{array}{lrr} & \int_{0}^{T}e^{-\kappa s}e^{\kappa (1-e^{-s})}f_{\kappa }(s)ds>0 & \end{array}$$

then, as κ<u, it automatically implies that


(2.19) $$\begin{array}{lrr} & \int_{0}^{T}\partial_{u}y_{u}(t)dt=\int_{0}^{T}e^{-us}e^{\kappa (1-e^{-s})}f_{\kappa }(s)ds>0. & \end{array}$$

To see this let $s^{*}$ be the time in [0,T] where the function $f_{\kappa }(s)$ crosses the x-axis. Then (2.18) implies that


$$\begin{array}{rl} & \int_{0}^{T}e^{-\kappa s}e^{\kappa (1-e^{-s})}f_{\kappa }(s)ds\\ & =\int_{0}^{s^{*}}e^{-\kappa s}e^{\kappa (1-e^{-s})}f_{\kappa }(s)ds+\int_{s^{*}}^{T}e^{-\kappa s}e^{\kappa (1-e^{-s})}f_{\kappa }(s)ds\\ & =\int_{0}^{s^{*}}e^{-\kappa s}e^{\kappa (1-e^{-s})}|f_{\kappa }(s)|ds-\int_{s^{*}}^{T}e^{-\kappa s}e^{\kappa (1-e^{-s})}|f_{\kappa }(s)|ds\\ & >0. \end{array}$$

Therefore, using that $e^{-us}\geq e^{-(u-\kappa )s^{*}}e^{-\kappa s}$ for $s\leq s^{*}$ and $e^{-us}\leq e^{-(u-\kappa )s^{*}}e^{-\kappa s}$ for $s\geq s^{*}$, we get


$$\begin{array}{rl} & \int_{0}^{T}e^{-us}e^{\kappa (1-e^{-s})}f_{\kappa }(s)ds\\ & =\int_{0}^{s^{*}}e^{-us}e^{\kappa (1-e^{-s})}|f_{\kappa }(s)|ds-\int_{s^{*}}^{T}e^{-us}e^{\kappa (1-e^{-s})}|f_{\kappa }(s)|ds\\ & \geq e^{-(u-\kappa )s^{*}}\left(\int_{0}^{s^{*}}e^{-\kappa s}e^{\kappa (1-e^{-s})}|f_{\kappa }(s)|ds-\int_{s^{*}}^{T}e^{-\kappa s}e^{\kappa (1-e^{-s})}|f_{\kappa }(s)|ds\right)\\ & >0. \end{array}$$

This shows that to prove the monotonicity result it suffices to prove (2.18), which is of course equivalent to proving the positivity of (2.11) for u=κ and $x_{0}=0$. As mentioned above, this positivity follows from (2.16) which is shown in Proposition 2.2. This completes the proof of the cDR monotonicity result for IFFL1.

## 3. Unconditional monotonicity of both dose response and cumulative dose response for incoherent feedforward loop 2

We now consider the IFFL2 system described by equations (1.7) and (1.8) . For this system, we can prove that the DR map, $u\mapsto y_{u}(t)$, is a monotonically increasing function of the input u, and hence, the same holds for the cDR map. A direct proof of this is provided in §3.1, while a more conceptual argument, based on the theory of monotone systems, is presented in §3.2. Although the former approach is simpler for this particular example, the latter is more generalizable to other examples.

### 3.1. Direct proof

In order to prove the monotonicity of the map u↦DR(u,T), it suffices to show that the partial derivative $\partial_{u}y_{u}(t)$ is non-negative for any u and t. Since $x_{u}(t)$ satisfies the linear ODE (1.7), we can solve for it explicitly to obtain


$$\begin{array}{r} x_{u}(t)=x_{0}e^{-\delta t}+\frac{u\alpha }{\delta }(1-e^{-\delta t}) \end{array}$$

which also implies that


(3.1) $$\begin{array}{lrr} & \partial_{u}x_{u}(t)=\frac{\alpha }{\delta }(1-e^{-\delta t})=\frac{x_{u}(t)-x_{0}e^{-\delta t}}{u}. & \end{array}$$

As $y_{u}(t)$ satisfies the ODE (1.8), we can differentiate it with respect to u to obtain an ODE for $\partial_{u}y_{u}(t)$ as


$$\begin{array}{rl} \partial_{u}{\dot{y}}_{u} & =\frac{\beta }{K+x_{u}(t)}-\frac{\beta u}{(K+x_{u}(t){)}^{2}}\partial_{u}x_{u}(t)-\gamma \partial_{u}y_{u}(t). \end{array}$$

Substituting $\partial_{u}x_{u}(t)$ from (3.1) and re-arranging we get


$$\begin{array}{rl} \partial_{u}{\dot{y}}_{u} & =\frac{\beta }{K+x_{u}(t)}-\frac{\beta u}{(K+x_{u}(t){)}^{2}}\left(\frac{x_{u}(t)-x_{0}e^{-\delta t}}{u}\right)-\gamma \partial_{u}y_{u}(t)\\ & =\frac{\beta }{K+x_{u}(t)}\left(1-\frac{x_{u}(t)}{K+x_{u}(t)}+\frac{x_{0}e^{-\delta t}}{K+x_{u}(t)}\right)-\gamma \partial_{u}y_{u}(t). \end{array}$$

Since K≥0, it is immediate that $\frac{x_{u}(t)}{K+x_{u}(t)}\leq 1$, and so we have the differential inequality


$$\begin{array}{r} \partial_{u}{\dot{y}}_{u}(t)\geq -\gamma \partial_{u}y_{u}(t). \end{array}$$

As $\partial_{u}y_{u}(0)=0$, by the comparison argument it follows that $\partial_{u}y_{u}(t)\geq 0$ for all t and u. This concludes the proof of the monotonicity result for IFFL2.

### 3.2. Monotone systems: an approach to show dose-response monotonicity of incoherent feedforward loop 2

Monotone systems were introduced in the pioneering work of Smale, Smith, Hirsch, Mallet-Paret, Sell and others [50–53]. They have the property that larger initial conditions give rise to larger state trajectories, where ‘larger’ is interpreted according to a specified order in the state variables. Special cases of monotonic systems are obtained when the order is a coordinatewise order. For example, in two dimensions, the ‘NorthEast’ (NE) is defined by saying that a point $\left(x_{2},y_{2}\right)$ is larger than a point $\left(x_{1},y_{1}\right)$ if both $x_{2}>x_{1}$ and $y_{2}>y_{1}$, that is, if it is to the ‘north’ and ‘east’ (higher, to the right) of the second point; similarly the ‘NorthWest’ order would be defined by asking that $x_{2}<x_{1}$ and $y_{2}>y_{1}$. (Note that these are ‘partial orders’ in the sense that two vectors may not be comparable: for example, neither (0,1) nor (1,0) is larger than the other in the NE order.) The generalization to external inputs and outputs [54] enabled the development of a network interconnection theory as well as leading to conclusions regarding the effect of inputs: for example, monotonic inputs result in monotonic transient behaviour [55]. This means that the DR (and thus also the cDR) will always be monotonic, for monotone systems.

These developments led to monotone systems playing a key role in analysing the global behaviour of dynamical systems in various areas of engineering as well as biology [56]. What makes monotone system theory so useful is that there are ways to check monotonicity without solving a set of differential equations $\dot{x}=f(x)$. For example, for the n-dimensional analogue of the NE order one requires that the off-diagonal terms of the Jacobian matrix of f should all be non-negative, and a similar condition holds if there are inputs. More generally, monotonicity with respect to some (not necessarily the NE) coordinatewise order requires that all loops in the interaction graph obtained from the off-diagonal terms of the Jacobian should have a net positive sign (see [57] for more discussion and examples).

When trying to apply monotone systems theory to our three paradigmatic examples IFFL1, IFFL2 and IFB, an obvious problem arises: these systems are not monotone with respect to any possible coordinatewise order, as IFFLs and negative feedback loops contradict the positive loop condition. However, in the case of the IFFL2 system, which we reproduce here for convenience:


$$\begin{array}{rl} \dot{x} & =\alpha u-\delta x\\ \dot{y} & =\beta \frac{u}{K+x}-\gamma y \end{array}$$

there is a Lie group of symmetries or ‘equivariances’ that preserve the output. These equivariances were the main object of study in the work in [8] on scale invariance, and were key to the analysis of an immunology model in [29]. Specifically, when K=0 the discussion in §1.6 showed that the equations do not change under the one-parameter Lie group of transformations (u,x,y)↦(pu,px,y), and in particular scaling u and x by the same constant does not alter the dynamics of y. This suggests introducing the new variable p:=u/x. Using the variables p and y, the equations become:


$$\begin{array}{rl} \dot{p} & =p(\delta -\alpha p)\\ \dot{y} & =\beta p-\gamma y\;. \end{array}$$

This is a monotone system, because the only off-diagonal term in the Jacobian is β>0. Therefore, the trajectories depend monotonically on $p(0)=u/x_{0}$, and hence also depend monotonically on u. A similar argument works for K>0, but now the FCD property fails and the equivariance will provide merely an embedding into a monotone system rather than an equivalence. Indeed, let p:=u/(K+x). Now the p equation is no longer decoupled from x. However, we can look at the following extended system (we add an equation $\dot{u}=0$ to convert the external input into a state variable):


$$\begin{array}{rl} \dot{u} & =0\\ \dot{x} & =\alpha u-\delta x\\ \dot{p} & =p\left(\frac{\delta x}{K+x}-\alpha p\right)\\ \dot{y} & =\beta p-\gamma y\;. \end{array}$$

The off-diagonal elements of the Jacobian are α, β, and $K\delta p/(K+x{)}^{2}$, all positive. Thus, the extended system is monotone, and therefore all variables, and in particular y, depend monotonically on u. This shows monotonicity of the DR, as claimed.

## 4. Conditional monotonicity of the cumulative dose response integral feedback

As our final example, we examine the IFB system defined by equations (1.9) and (1.10). Our objective is to show that, unlike the previously discussed incoherent feedforward loop systems, this negative feedback system can exhibit non-monotonic behaviour in the mapping u↦cDR(u,T). We also identify a condition under which this mapping remains monotonic. To simplify the analysis, we restrict our attention to the case K=0. If the property of monotonicity or non-monotonicity arises in this case, it will also persist for small positive values of K due to the parametric continuity of solutions.

We begin our analysis by simplifying the equations for the IFB system using scaling arguments in §4.1. Here, we also reformulate the dynamics in terms of the ‘error’, which is obtained by subtracting the input-independent steady state from y. Notably, the monotonicity properties of the original cDR map can be equivalently studied using the error cDR map. In §4.2, we prove that the partial derivative of the error cDR map, with respect to the input value u, is always positive at steady state, confirming that the steady-state cDR map is monotonic. For finite-time analysis, we establish a connection between the dynamics of the error and a ‘damped’ harmonic oscillator in §4.3. This connection allows us to derive a conditional monotonicity result for the error cDR map in §4.4. Furthermore, this result enables us to identify a finite range of time values for each input value u, within which the error cDR map may become non-monotonic. We leverage this insight to numerically illustrate this non-monotonicity in §4.5.

### 4.1. Simplifying the system

Recall the IFB equations (1.9) and (1.10) . We scale time by $\gamma^{-1}$ and the state values by a ratio to define


$$\begin{array}{r} \hat{x}(t)=x(\gamma^{-1}t)\;\text{and}\;\hat{y}(t)=\frac{\alpha }{\gamma }y(\gamma^{-1}t). \end{array}$$

Then, we can write this system as


$$\begin{array}{rl} \dot{\hat{x}}(t) & =\gamma^{-1}\dot{x}(\gamma^{-1}t)=\gamma^{-1}x(\gamma^{-1}t)(\alpha y(\gamma^{-1}t)-\delta )=\hat{x}(t)\left(\hat{y}(t)-\frac{\delta }{\gamma }\right)\\ \dot{\hat{y}}(t) & =\frac{\alpha }{\gamma^{2}}\left(\frac{\beta u}{x(\gamma^{-1}t)}-\gamma y(\gamma^{-1}t)\right)=\frac{\alpha \beta u}{\gamma^{2}\hat{x}(t)}-\hat{y}(t). \end{array}$$

Therefore, if we define


$$\hat{u}=\frac{\alpha \beta }{\gamma^{2}}u\;\text{and}\;p=\frac{\delta }{\gamma }$$

then $\left(\hat{x}(t),\hat{y}(t)\right)$ satisfies the ODEs


$$\begin{array}{rl} \dot{\hat{x}}(t) & =\hat{x}(t)(\hat{y}-p)\\ \dot{\hat{y}}(t) & =\frac{\hat{u}}{\hat{x}}-\hat{y}. \end{array}$$

with initial values $\hat{x}(0)=x(0)=x_{0}$ and $\hat{y}(0)=\frac{\alpha }{\gamma }y(0)=\frac{\alpha }{\gamma }\frac{\delta }{\alpha }=\frac{\delta }{\gamma }=p$. Henceforth, we shall drop the hats for notational convenience and suppose that the dynamics is given by


(4.1)x˙=x(y−p)(4.2)y˙=ux−y

with initial values $x(0)=x_{0}$ and y(0)=p. Let $\left(x_{u}(t),y_{u}(t)\right)$ be the solution of this initial value problem for t≥0.

Note that p is both the initial state and the steady-state value of the output variable $y_{u}(t)$. We shall reformulate the dynamics in terms of the ‘error’


$${\tilde{y}}_{u}(t)=y_{u}(t)-p$$

and variable $z_{u}(t)$ defined as


$$z_{u}(t)=\frac{u}{x_{u}(t)}.$$

Then, dynamics of $z_{u}(t)$ is given by


$$\begin{array}{r} {\dot{z}}_{u}(t)=-\frac{u}{x_{u}^{2}(t)}{\dot{x}}_{u}(t)=-\frac{u}{x_{u}(t)}(y_{u}(t)-p)=-z_{u}(t){\tilde{y}}_{u}(t). \end{array}$$

Hence, we can solve for $z_{u}(t)$ as


(4.3) $$\begin{array}{lrr} & z_{u}(t)=\frac{u}{x_{0}}e^{-\int_{0}^{t}{\tilde{y}}_{u}(s)ds} & \end{array}$$

and the dynamics of the error is given by


(4.4) $$\begin{array}{lrr} & {\dot{\tilde{y}}}_{u}(t)={\dot{y}}_{u}(t)=z_{u}(t)-y_{u}(t)=z_{u}(t)-p-{\tilde{y}}_{u}(t). & \end{array}$$

Henceforth, instead of examining the monotonicity of the original cDR map, we shall equivalently examine the monotonicity of the error cDR map given by


$$u\mapsto \int_{0}^{T}{\tilde{y}}_{u}(t)dt.$$

### 4.2. Steady-state analysis

We proved earlier that the steady state is stable. Thus, $\lim_{t\to \infty }\;{\tilde{y}}_{u}(t)=0$ and so $\lim_{t\to \infty }\;z_{u}(t)=p$. Therefore, expression (4.3) implies that


(4.5) $$\begin{array}{lrr} & \int_{0}^{\infty }{\tilde{y}}_{u}(t)dt=\log(u)-\log(px_{0}) & \end{array}$$

which is a monotonically increasing function of u. In particular, by differentiating with respect to u we obtain


(4.6) $$\begin{array}{lrr} & \int_{0}^{\infty }\partial_{u}{\tilde{y}}_{u}(t)dt=\frac{1}{u}>0. & \end{array}$$

If this positivity holds for any finite time-interval then we shall have the monotonicity of the error cDR map. We shall show that this monotonicity does not always hold and identify a sufficient condition under which it does. For this we shall connect this problem to a harmonic oscillator with a time-varying frequency.

### 4.3. Connection to a harmonic oscillator

Differentiating the error equation (4.4) with respect to t and using that ${\dot{z}}_{u}(t)=-z_{u}(t){\tilde{y}}_{u}(t)$ we get


$$\begin{array}{r} {\overset{"}{\tilde{y}}}_{u}(t)={\dot{z}}_{u}(t)-{\dot{\tilde{y}}}_{u}(t)=-z_{u}(t){\tilde{y}}_{u}(t)-{\dot{\tilde{y}}}_{u}(t) \end{array}$$

which means that the error ${\tilde{y}}_{u}(t)$ satisfies the equation for a ‘damped’ harmonic oscillator with a non-constant frequency:


(4.7) $$\begin{array}{lrr} & {\overset{"}{\tilde{y}}}_{u}(t)+{\dot{\tilde{y}}}_{u}(t)+z_{u}(t){\tilde{y}}_{u}(t)=0, & \end{array}$$

with initial conditions ${\tilde{y}}_{u}(0)=0$ and ${\dot{\tilde{y}}}_{u}(0)=z_{u}(0)-p=\frac{u}{x_{0}}-p$. Let us define the ‘frequency’ and Hamiltonian for this oscillator as


$$\omega (t)=\sqrt{z_{u}(t)}\;\text{and}\;H(t)=({\tilde{y}}_{u}(t){)}^{2}+{\left(\frac{{\dot{\tilde{y}}}_{u}(t)}{\omega (t)}\right)}^{2},$$

respectively. Then, their dynamics can be derived as


(4.8) $$\begin{array}{lrr} & \dot{\omega }(t)=\frac{1}{2\omega (t)}{\dot{z}}_{u}(t)=-\frac{1}{2\omega (t)}z_{u}(t){\tilde{y}}_{u}(t)=-\frac{{\tilde{y}}_{u}(t)}{2}\omega (t). & \end{array}$$

Using (4.7) we obtain


(4.9) $$\begin{array}{lrlr} & \dot{H}(t) & =2{\tilde{y}}_{u}(t){\dot{\tilde{y}}}_{u}(t)+2\frac{{\dot{\tilde{y}}}_{u}(t){\overset{"}{\tilde{y}}}_{u}(t)}{z_{u}(t)}-{\left(\frac{{\dot{\tilde{y}}}_{u}(t)}{z_{u}(t)}\right)}^{2}{\dot{z}}_{u}(t) & \\ & & =2{\tilde{y}}_{u}(t){\dot{\tilde{y}}}_{u}(t)-2\frac{{\dot{\tilde{y}}}_{u}(t)}{z_{u}(t)}({\dot{\tilde{y}}}_{u}(t)+z_{u}(t){\tilde{y}}_{u}(t))+\frac{({\dot{\tilde{y}}}_{u}(t){)}^{2}}{z_{u}(t)}{\tilde{y}}_{u}(t) & \\ & & =-2{\left(\frac{{\dot{\tilde{y}}}_{u}(t)}{\omega (t)}\right)}^{2}\left(1-\frac{{\tilde{y}}_{u}(t)}{2}\right). & \end{array}$$

Observe that the error ${\tilde{y}}_{u}(t)=y_{u}(t)-p$ in our adapting circuit goes to 0 as t→∞. Equation (4.9) shows that when this error is below 2, the Hamiltonian is decreasing. Note that the initial value of this Hamiltonian is


$$\begin{array}{r} H(0)=\frac{(u-px_{0}{)}^{2}}{ux_{0}}. \end{array}$$

This brings us to a proposition that shows that if this value is less than 4, then the error ${\tilde{y}}_{u}(t)$ remains below 2 at all times.

**Proposition 4.1** *Suppose that the following holds:*


(4.10) $$\begin{array}{lrr} & (u-px_{0}{)}^{2}\leq 4ux_{0}. & \end{array}$$

*Then we must have that* ${\tilde{y}}_{u}(t)\leq 2$ *for all* t≥0.

*Proof.* Condition (4.10) implies that H(0)≤4. Recall that ${\tilde{y}}_{u}(0)=0$. Let $t_{1}$ be the first time ${\tilde{y}}_{u}(t)$ reaches 2, i.e. ${\tilde{y}}_{u}(t_{1})=2$ and ${\tilde{y}}_{u}(t)<2$ for all $t<t_{1}$. Then due to (4.9) the Hamiltonian must be decreasing in the interval $\left[0,t_{1}\right]$, and so $H(t_{1})<H(0)\leq 4$. But this is a contradiction since ${\tilde{y}}_{u}(t_{1})=2$ and so by definition $H(t_{1})\geq 4$. Hence, $t_{1}=\infty$ which means that ${\tilde{y}}_{u}(t)\leq 2$ for all t≥0.∎

### 4.4. Conditional monotonicity result

Now let us substitute $z_{u}(t)$ from (4.3) into equation (4.4) to obtain


(4.11) $$\begin{array}{lrr} & {\dot{\tilde{y}}}_{u}(t)=\frac{u}{x_{0}}e^{-\int_{0}^{t}{\tilde{y}}_{u}(s)ds}-p-{\tilde{y}}_{u}(t). & \end{array}$$

Differentiating this with respect to u we get


$$\begin{array}{rl} \partial_{u}{\dot{\tilde{y}}}_{u}(t) & =\frac{1}{x_{0}}e^{-\int_{0}^{t}{\tilde{y}}_{u}(s)ds}-\frac{u}{x_{0}}e^{-\int_{0}^{t}{\tilde{y}}_{u}(s)ds}\int_{0}^{t}\partial_{u}{\tilde{y}}_{u}(s)ds-\partial_{u}{\tilde{y}}_{u}(t)\\ & =\frac{u}{x_{0}}e^{-\int_{0}^{t}{\tilde{y}}_{u}(s)ds}\left(\frac{1}{u}-\int_{0}^{t}\partial_{u}{\tilde{y}}_{u}(s)ds\right)-\partial_{u}{\tilde{y}}_{u}(t)\\ & =z_{u}(t)\left(\frac{1}{u}-\int_{0}^{t}\partial_{u}{\tilde{y}}_{u}(s)ds\right)-\partial_{u}{\tilde{y}}_{u}(t). \end{array}$$

This shows that if we define β(t) as


$$\begin{array}{r} \beta (t)=1-u\int_{0}^{t}\partial_{u}{\tilde{y}}_{u}(s)ds, \end{array}$$

then β(t) also satisfies the following second-order ODE for the damped harmonic oscillator (4.7) with initial conditions β(0)=1 and $\dot{\beta }(0)=0$. To prove the monotonicity we need to show that


$$\int_{0}^{t}\partial_{u}{\tilde{y}}_{u}(s)ds\geq 0$$

which is equivalent to proving that


(4.12) $$\begin{array}{lrr} & \beta (t)\leq 1\;\text{for all}\;t\geq 0. & \end{array}$$

Again we can definite the Hamiltonian as


(4.13) $$\begin{array}{lrr} & H_{\beta }(t)=(\beta (t){)}^{2}+{\left(\frac{\dot{\beta }(t)}{\omega (t)}\right)}^{2} & \end{array}$$

and it will have the same dynamics as before:


(4.14) $$\begin{array}{lrr} & \dot{H_{\beta }}(t)=-2{\left(\frac{\dot{\beta }(t)}{\omega (t)}\right)}^{2}\left(1-\frac{{\tilde{y}}_{u}(t)}{2}\right) & \end{array}$$

which we can also write as


(4.15) $$\begin{array}{lrlr} & \dot{H_{\beta }}(t) & =-H_{\beta }(t)(2-{\tilde{y}}_{u}(t))+(\beta (t){)}^{2}(2-{\tilde{y}}_{u}(t)). & \end{array}$$

We now come to our main result for the IFB example, which proves monotonicity of the error cDR under condition (4.10).

**Proposition 4.2** *Suppose that condition* (4.10) *holds. Then, the map*


$$\begin{array}{r} u\mapsto \int_{0}^{T}{\tilde{y}}_{u}(t)dt \end{array}$$

*is monotonically increasing for any* T>0.

*Proof*. When (4.10) holds, we have that the error ${\tilde{y}}_{u}(t)\leq 2$ for all t≥0 due to proposition 4.1. This fact along with (4.14) implies that $H_{\beta }(t)\leq H_{\beta }(0)=1$ for t≥0. Therefore, (4.12) holds which proves the required monotonicity property.∎

### 4.5. Numerical illustration of non-monotonicity

When condition (4.10) does not hold, we do not have any analytical approach for checking the monotonicity property. Therefore, we need to rely on numerical simulations. For any given u, the monotonicity condition (4.12) can fail at any t≥0. However, as we cannot check this condition for all t≥0, we first show that if this monotonicity fails then it has to fail in the finite time - interval $\left[0,T_{u}\right]$ where $T_{u}$ is defined by


(4.16) $$\begin{array}{lrr} & T_{u}=\inf\{t\geq 0:H(t)\leq 4\;\text{and}\;H_{\beta }(t)\leq 1\}. & \end{array}$$

Note that $T_{u}$ is finite because both H(t) and $H_{\beta }(t)$ are non-negative quantities converging to zero as t→∞. This is due to stability and the fact that (4.6) implies that β(t)→0 as t→∞.

**Proposition 4.** $T_{u}$ *be the finite time defined by* (4.16)*. Then, we must have* β(t)≤1 *for all* $t\geq T_{u}$ *which is the same as saying that* $u\int_{0}^{t}\partial_{u}y(s)ds\geq 0$ *for all* $t\geq T_{u}$.

*Proof.* Pick a small ϵ>0 and define


$$\begin{array}{r} T_{u}^{\epsilon }=\inf\{t\geq 0:H(t)\leq 4-\epsilon \;\text{and}\;H_{\beta }(t)\leq 1-\epsilon \}. \end{array}$$

Note that for the same reason that $T_{u}$ is finite, $T_{u}^{\epsilon }$ must also be finite. Moreover, as ϵ decreases the set $\left\{t\geq 0:H(t)\leq 4-\epsilon \;\text{and}\;H_{\beta }(t)\leq 1-\epsilon \right\}$ gets bigger and consequently its infimum, which is $T_{u}^{\epsilon }$, gets smaller. Therefore, $T_{u}^{\epsilon }$ decreases monotonically as ϵ decreases and since H(t) and $H_{\beta }(t)$ are continuous functions of time we must have


(4.17) $$\begin{array}{r} \lim_{\epsilon ↘0}T_{u}^{\epsilon }=T_{u}. \end{array}$$

By the definition of $T_{u}^{\epsilon }$ we have that $H(T_{u}^{\epsilon })<4$ which implies that ${\tilde{y}}_{u}(T_{u}^{\epsilon })<2$. Let $t_{1}^{\epsilon }$ be the first time after $T_{u}^{\epsilon }$ such that ${\tilde{y}}_{u}(t_{1}^{\epsilon })=2$ and consequently $H(t_{1}^{\epsilon })\geq 4$. On the open interval $\left(T_{u}^{\epsilon },t_{1}^{\epsilon }\right)$, H(t) must be decreasing due to (4.9) and so we should have $H(t_{1}^{\epsilon })\leq H(T_{u}^{\epsilon })$. However, this leads to a contradiction because $H(T_{u}^{\epsilon })<4\leq H(t_{1}^{\epsilon })$. Therefore, $t_{1}^{\epsilon }=\infty$ which means that ${\tilde{y}}_{u}(t)<2$ for all $t\geq T_{u}^{\epsilon }$. This also means that $H_{\beta }(t)$ is decreasing in the interval $\left[T_{u}^{\epsilon },\infty \right)$ due to (4.14). Since $H_{\beta }(T_{u}^{\epsilon })<1$ this implies that β(t)<1 for all $t\geq T_{u}^{\epsilon }$. Using the continuity of β and the limit (4.17) we can conclude that β(t)≤1 for all $t\geq T_{u}$. This completes the proof of this proposition.∎

**Figure 8.**
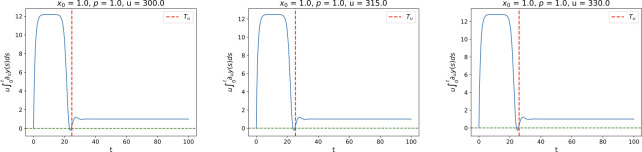
Plot of the map $t\mapsto u\int_{0}^{t}\partial_{u}y(s)ds$ for three values of u. Note that for each u, we have $u\int_{0}^{t}\partial_{u}y(s)ds\geq 0$ for all $t\geq T_{u}$.

Figure 8 illustrates this proposition for three values of u. One can observe that $u\int_{0}^{t}\partial_{u}y(s)ds\geq 0$ holds for all $t\geq T_{u}$ for each u-value.

**Figure 9.**
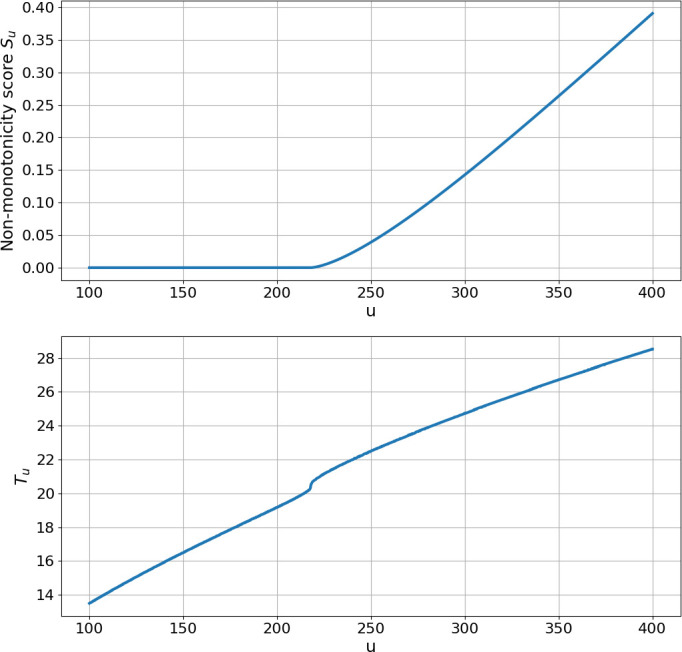
Plot of the maps u↦S(u) and u↦T(u) with $x_{0}=p=1.0$.


**Figure 10.**
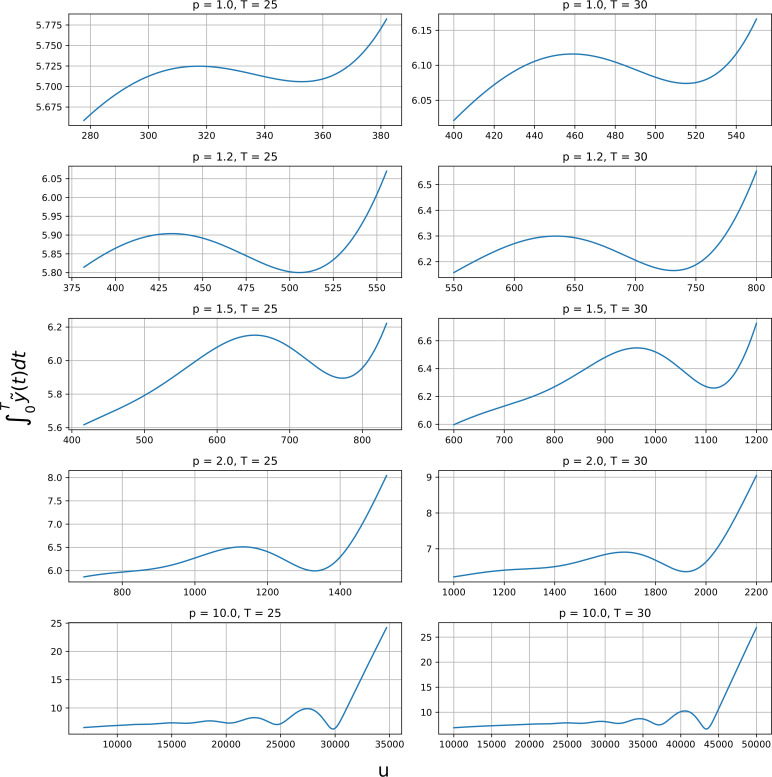
Plot of the map $u\mapsto \int_{0}^{T}{\tilde{y}}_{u}(t)dt$ for some values of p and T. Note that this map exhibits non-monotonicity in these cases.

We can now numerically solve the system $\left(z_{u}(t),y_{u}(t),\beta (t)\right)$ in the interval $\left[0,T_{u}\right]$ and check if β(t) exceeds 1 or not. For any t such that β(t)>1 we would have $\int_{0}^{t}\partial_{u}{\tilde{y}}_{u}(s)ds<0$, which would imply non-monotonicity. For any given u we define the non-monotonicity score as


(4.18) $$\begin{array}{lrr} & S(u)=\int_{0}^{T_{u}}{\left(\beta (t)-1\right)}^{+}dt=u\int_{0}^{T_{u}}{\left(\int_{0}^{t}\partial_{u}{\tilde{y}}_{u}(s)ds\right)}^{-}dt, & \end{array}$$

where $a^{+}$ (resp. $a^{-}$) denotes the positive (resp. negative) part of a. In figure 9 we plot the non-monotonicity score S(u) and the time $T_{u}$ for a range of u-values for $x_{0}=p=1$. Notice that $T_{u}$ is monotonically increasing with u, while the non-monotonicity score S(u) stays at zero untill u≈220 and then it gradually starts increasing. This shows that for higher values of u the map

$$u\mapsto \int_{0}^{T}{\tilde{y}}_{u}(t)dt$$

is always non-monotonic for some T which will increase as u increases. Figure 10 illustrates this non-monotonicity for some values of p and T.

## 5. Conclusions

We introduced the notion of cDR, and went on to show mathematically that both the incoherent feedback loop IFFL1 and IFFL2 motifs can only produce monotonic cDRs, even if (for IFFL1) the DR itself may be non-monotonic. On the other hand, we also established conditions under which the IFB mechanism can produce a non-monotonic cDR.

Leaving aside linear systems, which can never lead to non-monotonic (or, for that matter, any nonlinear) cDR, the motifs that we analysed are considered the simplest paradigms for adaptation in biology [7,8,43]. The concepts introduced here are broadly applicable, even if results were established for idealized two-variable systems that adapt perfectly. They provide a foundation for developing a more comprehensive mathematical theory that can qualitatively characterize cDR maps in more complex, multi-variable systems. One particularly interesting direction would be to study how network interconnections (such as cascades) of these motifs can preserve the qualitative properties of cDR’s. Another is to include scenarios with non-ideal behaviours, such as species dilution, saturation in reaction rates and resource competition, as well as imperfect adaptation.

One may view our study of the cDR properties as an addition to the toolkit of mathematical methods for model discrimination and invalidation, in a spirit similar to the work in [58] that infers the existence of IFFLs or negative feedback loops when time responses are non-monotonic, or to the work in [10] on using periodic inputs to rule out IFFLs as the basis of adaptation. In such a role, one can perform dose-dependent experiments and on the basis of plotted cDR’s, eliminate systems whose structure is not consistent with an observed non-monotonic cDR. Conversely, one could ask how to introduce new variables or otherwise modify a model to match such qualitative behaviours.

## Data Availability

The simulation code uses Matlab and Python, and all parameters are explained in the paper. A spreadsheet with data from Trendle *et al* [59] as well as all code are available from the Zenodo repository [60].
